# Terminal modifications independent cell-free RNA sequencing enables sensitive early cancer detection and classification

**DOI:** 10.1038/s41467-023-44461-y

**Published:** 2024-01-02

**Authors:** Jun Wang, Jinyong Huang, Yunlong Hu, Qianwen Guo, Shasha Zhang, Jinglin Tian, Yanqin Niu, Ling Ji, Yuzhong Xu, Peijun Tang, Yaqin He, Yuna Wang, Shuya Zhang, Hao Yang, Kang Kang, Xinchun Chen, Xinying Li, Ming Yang, Deming Gou

**Affiliations:** 1 College of Life Sciences and Oceanography, Shenzhen University, Shenzhen, Guangdong China; 2 College of Physics and Optoelectronic Engineering, Shenzhen University, Shenzhen, Guangdong China; 3 Department of Clinical Laboratory, Peking University Shenzhen Hospital, Shenzhen, Guangdong China; 4 Department of Clinical Laboratory, People’s Hospital of Bao’an Shenzhen, Shenzhen, Guangdong China; 5 Department of Tuberculosis, The Fifth People’s Hospital of Suzhou, Suzhou, Jiangsu China; 6 Surgical Department, General Hospital of Ningxia Medical University, Yinchuan, Ningxia China; 7 School of Basic Medical Sciences, Ningxia Medical University, Yinchuan, Ningxia China; 8 Department of Clinical Laboratory, The Second People’s Hospital of Shenzhen, Shenzhen, Guangdong China; 9 College of Medicine, Shenzhen University, Shenzhen, Guangdong China; 10 Shenzhen Geneups Biotechnology Co., Shenzhen, Guangdong China

**Keywords:** RNA sequencing, Cancer screening, Next-generation sequencing

## Abstract

Cell-free RNAs (cfRNAs) offer an opportunity to detect diseases from a transcriptomic perspective, however, existing techniques have fallen short in generating a comprehensive cell-free transcriptome profile. We develop a sensitive library preparation method that is robust down to 100 µl input plasma to analyze cfRNAs independent of their 5’-end modifications. We show that it outperforms adapter ligation-based method in detecting a greater number of cfRNA species. We perform transcriptome-wide characterizations in 165 lung cancer, 30 breast cancer, 37 colorectal cancer, 55 gastric cancer, 15 liver cancer, and 133 cancer-free participants and demonstrate its ability to identify transcriptomic changes occurring in early-stage tumors. We also leverage machine learning analyses on the differentially expressed cfRNA signatures and reveal their robust performance in cancer detection and classification. Our work sets the stage for in-depth study of the cfRNA repertoire and highlights the value of cfRNAs as cancer biomarkers in clinical applications.

## Abstract (web summary)

Cell free RNA is a potentially valuable resource to detect cancer, however, its low concentration in plasma can limit usefulness. Here, the authors devise a library preparation method from 100ul of plasma, and apply to multiple cancer types to detect and classify cancer patients

## Introduction

Early diagnosis of malignancy offers the greatest opportunity to improve long-term patient survival by increasing the likelihood of cure^1^. Utilization of liquid biopsies for cancer detection is rapidly gaining prominence in clinical practice^2^. Although cell-free DNA-based assays interrogating somatic mutations^3^, fragmentation patterns^4,5^, and methylation signatures^6–8^ have shown high specificity in detecting and localizing cancer, complementing them with transcriptomic information will improve their sensitivity in diagnosing cancer at its earliest stage. Cell-free RNAs (cfRNAs) represent a landscape of extracellular transcripts and thus provide extensive clinical value^9,10^. Alterations in cellular RNA expression are a dynamic process that can serve as an indicator of disease^11^. Overexpression of specific tumor-associated transcripts can result in amplification of tumor-derived cfRNA signals in the blood^12^. The release of cfRNAs into the bloodstream is not solely through cell death but can also occur through dynamic signaling processes of extracellular vesicles^13^, expanding the opportunities to detect cancer in individuals with limited levels of circulating tumor DNA.

Over the past decades, the characterization of cfRNAs has mostly focused on microRNAs (miRNAs)^14–16^, which have limited tissue or disease specificity^17^. Recently, there has been a rising interest in the study of messenger RNAs (mRNAs), which have been shown to possess tissue and cancer subtype specificity^12^ as well as cell types of origin specificity^18^. mRNAs can also distinguish pre-malignant conditions of cancer^19^ and evaluate pathophysiological alterations in the brain^20^. Moreover, mRNA signatures have demonstrated value in obstetric care through early prediction of preeclampsia^21–24^. However, miRNA and mRNA account for only a small fraction of the cfRNA repertoire. The potential value of other kinds of cfRNAs in plasma remains elusive. There are two major barriers to comprehensive cfRNA profiling. First, large volumes of plasma (up to 2 ml) are required because the recovery rate of cfRNAs is typically low. Second, cfRNAs are severely fragmented, resulting in diverse terminal modifications^25^. However, conventional adapter ligation-based library preparation workflows are only sensitive to RNA fragments with a 5’-phosphate (5’-P) and 3’-hydroxyl (3’-OH) group^26,27^. Therefore, innovative cfRNA profiling technologies that can efficiently handle low abundance and highly fragmented samples are urgently needed.

Here, we develop Splint Ligation and Phosphate-independent RNA Sequencing (SLiPiR-seq), a sensitive library preparation method that remains reliable with as little as 100 µl of input plasma and allows profiling of cfRNAs independent of their 5’-end phosphorylation, coupled with a bioinformatics analysis pipeline (Supplementary Fig. 1) for the discovery and absolute quantification of non-canonical small RNA species in plasma. To verify the clinical feasibility of SLiPiR-seq, we conduct transcriptome-wide characterizations in patients with various cancer types and cancer-free donors, demonstrating its ability to detect transcriptomic changes occurring in early-stage tumors. We further carry out machine learning analyses and establish multiple models to detect and localize different types of cancers, highlighting the strong potential of cfRNA signatures for future clinical applications.

## Results

### Technology optimizations of SLiPiR-seq

**Fig. 1:**
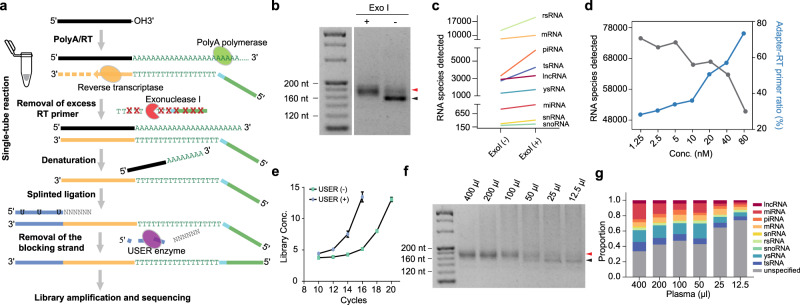
Technology optimizations of SLiPiR-seq. **a** Schematic representation of the SLiPiR-seq workflow. **b** Gel electrophoresis showing the effect of ExoI treatment on the final library size. Black arrow indicates the library size derived from the ligation between RT primer and adapter. Red arrow indicates the correct cfRNA library size. **c** Effect of ExoI treatment on the number of cfRNA species detected (RPM > 0) from nine RNA types. **d** Effect of different concentration of RT primer (from 1.25 nM to 80 nM) on the number of cfRNA species detected and adapter-RT primer reads ratio. **e** Effect of USER enzyme treatment on library amplification efficiency. Library concentrations (ng/µl) of different amplification cycles are shown. Points represent mean of three technical replicates. Error bars indicate standard deviation (SD). **f** Gel electrophoresis showing the size of final libraries produced by different starting input volumes of plasma (from 12.5 µl to 400 µl). **g** Proportion of different kinds of cfRNAs across different starting input volumes of plasma. For all graphs, *N* = 3 technical replicates were performed for all conditions, and the mean of the replicates was calculated. Source data are provided as a Source Data file.

To perform library preparation for highly fragmented cfRNAs, we optimized the S-Poly(T) Plus method that was previously developed for high-throughput small RNA profiling^28–30^. In brief, the input cfRNA was subjected to 3’-end polyadenylations along with reverse transcription (RT) in a one-step reaction (Fig. 1a). A custom synthetic RT primer consisting of oligo(dT), sample barcode, and sequencing adapter sequences was designed. cDNA with a 5’-adapter was obtained after the polyadenylations/RT reaction. The excess primer was then depleted using exonuclease I (ExoI). This is a critical step as any remaining RT primer can lead to adapter-primer ligation (~160 nt) (Fig. 1b), which would significantly impact the ratio of informative reads and the number of RNAs detected (Fig. 1c, Supplementary Fig. 2a–c). Consistently, similar results were observed upon the addition of excessive RT primer (Fig. 1d, Supplementary Fig. 2d, e). Subsequently, a double-stranded adapter with 3’-overhanding degenerate nucleotides was annealed to the 3’ end of cDNA by splint ligation (Fig. 1a). The randomized bases on the degenerate extension can transiently hybridize to cDNA^31,32^. The blocking strand of the adapter was then removed by excision of uracil residues using USER enzyme, followed by library amplification and sequencing. Removal of the blocking strand can significantly improve library amplification efficiency by facilitating primer binding (Fig. 1e, Supplementary Fig. 3a–d). To reduce RNA/DNA loss during library preparation, we further optimized SLiPiR-seq protocol by making the entire workflow compatible within a single tube.

To generate stable and reproducible transcriptome profiles, we extensively benchmarked SLiPiR-seq regarding plasma input volumes (from 12.5 µl to 400 µl) and the impact of various pre-analytical conditions (i.e., blood standing time before plasma separation and number of freeze/thaw cycles of the plasma). We found that the lower limit of plasma input for reliable SLiPiR-seq results is 100 µl (Fig. 1f, Supplementary Fig. 3e, f), significantly lower than the minimum requirement for most commercially available small RNA library preparation kits. The ratio of unspecified reads increased as the starting plasma volume decreased (Fig. 1g). We observed no significant difference in the recovery of total cfRNAs among plasma samples separated after 3, 6, and 9 hours of blood collection (3 h vs. 6 h *p* = 0.291; 3 h vs. 9 h *p* = 0.189) (Supplementary Fig. 4a). Furthermore, our results showed that cfRNAs remain stable upon one freeze/thaw cycles of plasma (*p* = 0.237) (Supplementary Fig. 4b). However, the informative reads were significantly reduced upon more freeze/thaw cycles (*p* < 0.001). Correlation analysis suggested that long noncoding RNAs (lncRNAs), mRNAs, miRNAs, small nuclear RNAs (snRNAs), small nucleolar RNAs (snoRNAs), and transfer RNA-derived small RNAs (tsRNAs) are stable in plasma under different pre-analytical conditions (Supplementary Fig. 4c, d). Taken together, we determined to separate the plasma within 3 hours of blood collection and to preserve 200 µl of aliquoted plasma by freezing it until SLiPiR-seq was performed to ensure one freeze/thaw cycle.

### Technology assessments of SLiPiR-seq

**Fig. 2:**
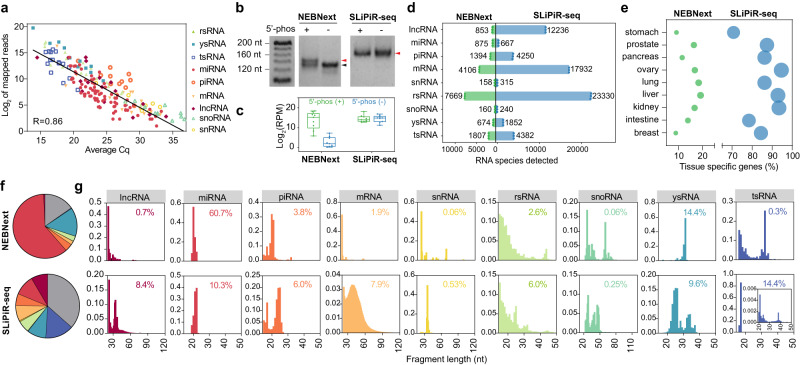
Technology assessments of SLiPiR-seq. **a** Linear models fitted to log_2_ RPM normalized sequencing reads profiled by SLiPiR-seq and Ct value measured by qPCR. The Pearson correlation coefficient (*R*) value is shown on the bottom left. **b**, **c** Gel electrophoresis (**b**) and the detected clean reads (**c**) of cfRNA libraries generated by NEBNext and SLiPiR-seq using the mixture of nine synthetic small RNAs with or without a 5’ phosphate group. The data point represents individual synthetic RNA. The extremes of boxes define the upper and lower quartiles, and the center lines define the median. Whiskers indicate 1.5× interquartile range (IQR). **d** The number of different types of RNAs detected from human plasma at a threshold of rpm > 0 by the two methods. Mean and SD of three technical replicates are shown. **e** Percentage of tissue-specific genes detected by NEBNext and SLiPiR-seq. **f** Composition of sequencing reads from different types of RNAs in NEBNext and SLiPiR-seq. Gray color indicates unspecified sequences. **g** Density plots showing the fragment size distribution for each type of RNA. The percentage values shown in the upper right corner are corresponding to the RNA proportion in (f). Source data are provided as a Source Data file.

To assess whether the sequencing results of SLiPiR-seq can accurately represent the molecular characteristics of the plasma transcriptome, we compared it to quantitative real-time PCR (qPCR), the gold standard technique for RNA quantification. We randomly selected varying expression levels of cfRNAs detected by SLiPiR-seq for comparison. Primer design was based on our previously described S-Poly(T) Plus strategy^28,29^. Notably, we observed a high concordance (*R* = 0.86) between read counts profiled by SLiPiR-seq and Ct values measured by qPCR (Fig. 2a, Supplementary Fig. 5, Supplementary Data 1), suggesting that SLiPiR-seq can accurately profile the plasma transcriptome.

Taking advantage of the splint ligation, we hypothesized that SLiPiR-seq could be used to profile cfRNAs independently of their 5’-end phosphorylation, thereby providing a broader coverage of the cfRNA repertoire. To test this hypothesis, we compared SLiPiR-seq with an adapter ligation-based method (NEBNext Small RNA Library Prep Set, NEBNext) using synthetic small RNAs with or without a 5’-P group. Our findings revealed that SLiPiR-seq can reliably construct a sequencing library regardless of the presence or absence of a 5’-P group. However, adapter ligation of NEBNext failed when using RNAs lacking a 5’-P group (Fig. 2b). Consistently, sequencing read counts generated by SLiPiR-seq showed no difference between the two groups given the same amount of library input, whereas NEBNext exhibited sensitivity only when the 5’-end phosphorylation was present (Fig. 2c, Supplementary Data 2).

We subsequently compared the two library preparation methods to evaluate their capacity to detect cfRNAs in human plasma samples. SLiPiR-seq detected 3.68 times more total RNA species than NEBNext (65204 vs. 17696) with 20 million clean reads (Fig. 2d). Specifically, 17932 mRNA and 12236 lncRNA species were detected by SLiPiR-seq, which were 4.37 and 14.34 times more than NEBNext, respectively. More importantly, by performing a cross-analysis with tissue-specific genes of various tissues from the Human Protein Atlas database, we discovered that a considerable number of mRNAs detected by SLiPiR-seq showed tissue-specific elevated expression patterns (Fig. 2e, Supplementary Data 2). Compared to NEBNext, SLiPiR-seq displayed a substantially increased proportion of mRNAs (7.9% vs. 1.9%) and lncRNAs (8.4% vs. 0.7%) (Fig. 2f). In addition, the fragment size of mRNAs (NEBNext: *α* = 3.32, *β* = 0.10; SLiPiR-seq: *α* = 10.22, *β* = 0.24, fitted to Gamma distribution) and lncRNAs (NEBNext: *α* = 6.29, *β* = 0.21; SLiPiR-seq: *α* = 12.07, *β* = 0.35) exhibited smoother distribution and broader coverage in SLiPiR-seq, as suggested from the kurtosis values (mRNA: 13.22 for NEBNext vs. 11.25 for SLiPiR-seq; lncRNA: 30.58 for NEBNext vs. 14.92 for SLiPiR-seq) (Fig. 2g). This finding was further validated by analyzing the fragment length of the twenty most highly expressed mRNA and lncRNA species (Supplementary Fig. 6a, b). Exonic mRNA and lncRNA sequences detected by both methods were derived from the sense orientation rather than the antisense orientation (Supplementary Fig. 6c), suggesting successful recovery of transcript. Our findings also indicated that SLiPiR-seq detected a broader coverage of the 3’ end of mRNAs than NEBNext (Supplementary Fig. 6d, e).

miRNAs emerged as the predominant RNA type (60.7%) in NEBNext results (Fig. 2f), consistent with the fact that plasma miRNAs are in the canonical form with 5’-P and 3’-OH termini^33^. Although miRNAs accounted for a lower proportion (10.3%) of total cfRNAs in SLiPiR-seq, the expression levels of miRNAs detected by both methods were strongly correlated (*R* = 0.938, Supplementary Fig. 7). We also observed low correlations for the expression levels of other RNA types between the two methods, because SLiPiR-seq identified a greater number of RNA species than NEBNext. Together, these observations suggest that SLiPiR-seq holds great promise to uncover the biological implications of diseases in plasma, given its enhanced detection of more diverse and integral RNA species associated with gene expression and regulation.

### Characterizations of tsRNAs, rsRNAs, and ysRNAs

**Fig. 3:**
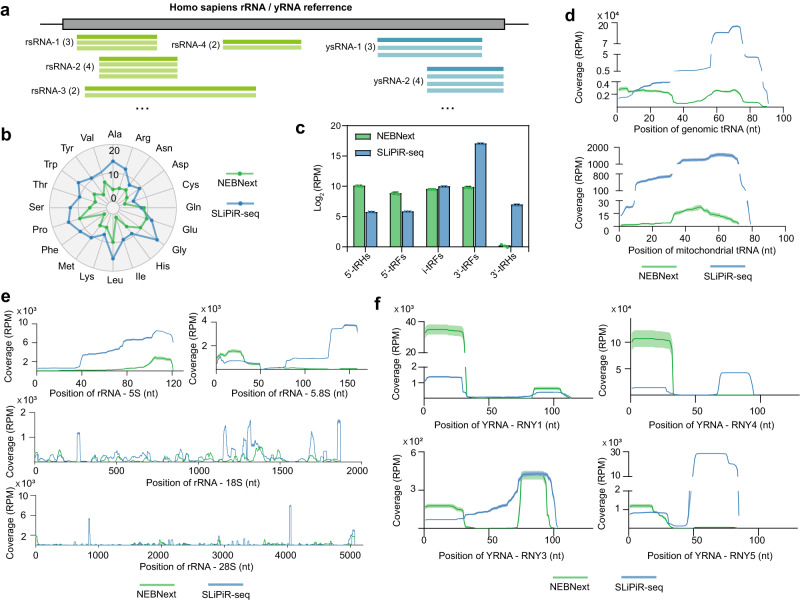
Characterization of tsRNAs, rsRNAs and ysRNAs. **a** Schematic diagram for the characterization of rsRNAs and ysRNAs. The numbers in the bracket represent read counts of RNAs. **b** Radar plot showing the corresponding amino acids of detected tsRNA in NEBNext and SLiPiR-seq results. The numbers on the radius represent log_2_RPM values. **c** Structural composition of tsRNA species identified by the two methods. tRHs refers to tRNA-halves, and tRFs refers to tRNA-derived fragments. i-tRFs refers to internal tRNA-derived fragments. Mean and SD of three technical replicates are shown. **d**–**f** Genome browser visualization of read coverage profiles of tsRNA mapped to Homo sapiens genomic tRNA and mitochondrial tRNA (**d**), rsRNA mapped to Homo sapiens ribosomal RNA (28 S, 18 S, 5.8 S, 5 S) (**e**), and ysRNA mapped to Homo sapiens Y RNA (RNY1, RNY3, RNY4, and RNY5) (**f**). The solid curves indicate mean RPM values (Mean), and the shadow represents error bars (SD). Source data are provided as a Source Data file.

Intrigued by the abundant expression of tsRNAs (14.4%), ribosomal RNA-derived small RNAs (rsRNAs, 6.0%), and Y RNA-derived small RNAs (ysRNAs, 9.6%) revealed by SLiPiR-seq (Fig. 2f) and recognizing the absence of publicly available reference genome for rsRNA and ysRNA, we purposed to establish a reference genome for cfRNAs aligned to rRNAs and Y RNAs. We mapped the small RNAs to the parent rRNAs (28 S, 18 S, 5.8 S, and 5 S rRNA) and Y RNAs (RNY1, RNY3, RNY4, and RNY5) and extracted unique sequences from the outputs of alignment to ensure a complete annotation of each rsRNA and ysRNA (Fig. 3a). From all the samples included in this study, we detected a total of 45397 rsRNA and 2664 ysRNA unique sequences (Supplementary Data 3). Together with 28,824 unique tsRNA sequences acquired from an online tsRNA database^34^, we used a custom Python script to call the read counts of tsRNAs, rsRNAs, and ysRNAs. This script only tallies a fragment if it aligns perfectly with a sequence in the FASTA file (Fig. 3a).

Next, we analyzed the differences between SLiPiR-seq and NEBNext in characterizing tsRNAs, rsRNAs, and ysRNAs. Compared to NEBNext, the number of amino acids corresponding to the parent tRNAs of tsRNAs was significantly increased in SLiPiR-seq (Fig. 3b). Furthermore, our results showed that SLiPiR-seq identified a higher overall sequencing depth and broader coverage of the 3’ end than NEBNext, because RNA fragments resulting from 3’ end cleavage are more likely to lack a 5’-P group. For example, SLiPiR-seq displayed increased read counts for tsRNAs starting at the 3’end and ending at the anticodon position (3’-tRHs) or ending before the anticodon position (3’-tRFs) (Fig. 3c, d)^35^. This finding was particularly evident for the 3’end of 5.8 S rRNA, RNY4, and RNY5, which were undetectable using NEBNext (Fig. 3e, f). These findings highlight the potential of SLiPiR-seq in discovering underexplored small RNAs in blood.

### Identification of cfRNA signatures for lung cancer using SLiPiR-seq

**Fig. 4:**
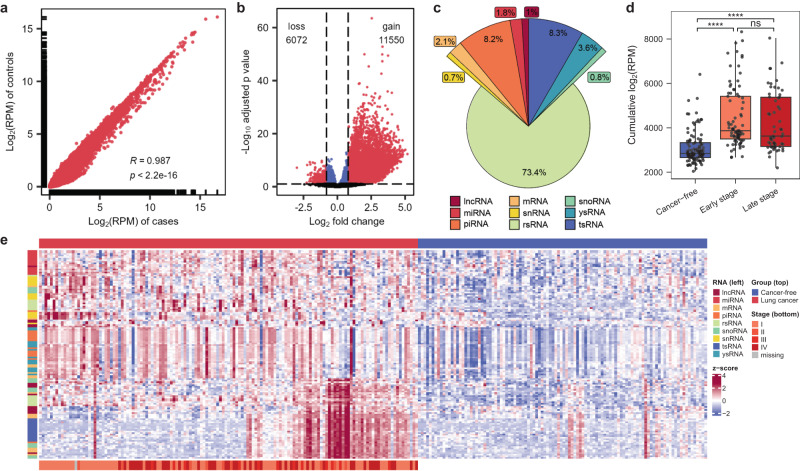
Identification of differentially expressed cfRNAs using SLiPiR-seq. **a** Transcriptome-wide Pearson’s correlation of log_2_ transformed RPM normalized reads of all detected cfRNAs between cases and controls. Two-sided *t* statistic was used to determine the *p* value based on Pearson correlation coefficient. *p* < 2.2e-16 represents a value that is extremely close to zero. **b** Volcano plot of DE cfRNAs from LC patients (cases, *N* = 139) versus cancer-free donors (controls, *N* = 106). Red dots indicate cfRNAs at Benjamini–Hochberg false discovery rate (BH-FDR) < 0.1 (Wald test, two-sided *P* values) and absolute log_2_ fold change >0.8. **c** Pie plot showing type distributions of DE cfRNAs. **d** Cumulative sum of the normalized reads of all upregulated (fold change >0) DE cfRNAs between early-stage LC patients (*N* = 81), late-stage LC patients (*N* = 57), and controls (*N* = 106) (Welch’s *t* test, two-sided). Early-stage LC patients vs. controls *p* = 7.10 × 10^−13^; Late-stage LC patients vs. controls *p* = 4.82 × 10^−8^; Early-stage vs. late-stage *p* = 0.321. The extremes of boxes define the upper and lower quartiles, and the center lines define the median. Whiskers indicate 1.5× interquartile range (IQR). **e** Heatmap of the top 15 DE cfRNAs of each type between cases and controls (Euclidean distance, Ward D2 clustering for row). Z score normalization was performed on the log_2_ transformed RPM normalized reads. Source data are provided as a Source Data file.

To investigate whether SLiPiR-seq can detect differentially expressed (DE) cfRNAs in cancer, we retrospectively profiled a discovery cohort containing plasma samples from 139 lung cancer (LC) patients (cases) and 106 cancer-free individuals (controls). Transcriptome-wide comparison of normalized sequencing reads between the two cohorts showed a high Pearson’s correlation (*R* = 0.987, *p* < 2.2 × 10^−16^), indicating that SLiPiR-seq has robust reproducibility (Fig. 4a, Supplementary Fig. 8). We identified 17622 DE cfRNAs (11550 gain and 6072 loss, adjusted *p* value < 0.1) between cases and controls based on a negative binomial model of fragment counts (Fig. 4b, Supplementary Fig. 9, Supplementary Data 4). The majority of DE cfRNAs have increased expression in cancer patients, consistent with the fact that more cfRNAs are released into the circulation in the context of cancer. The three most abundant categories of DE cfRNAs are rsRNAs (73.4%), tsRNAs (8.3%), and piwi-interacting RNAs (piRNAs) (8.2%) (Fig. 4c).

To enhance computational efficiency, we reduced our dataset by filtering out non-DE cfRNAs, followed by removal of cfRNAs with low expression levels (mean read counts <10) and minimal differences between cases and controls (log_2_ fold change <0.8). Next, we compared the sum of reads of the remaining DE cfRNAs between early-stage LC patients (stage I and II, *N* = 81), late-stage LC patients (stage III and IV, *N* = 57), and controls. We observed a significant increase in the cumulative expression level of DE cfRNAs in early-stage LC patients compared to controls (*p* = 7.10 × 10^−13^), and no difference (*p* = 0.321) between early and late-stage LC patients (Fig. 4d, Supplementary Fig. 10). Hierarchical cluster analysis using the top fifteen DE cfRNAs of each type revealed a higher level of DE cfRNAs in cancer patients than in cancer-free controls (Fig. 4e). As expected, no evident differences in the expression profiles of these cfRNAs between patients with different cancer stages were observed in the heatmap. These results indicate that SLiPiR-seq can detect aberrant transcriptional events that occur early in tumor pathogenesis, enabling SLiPiR-seq as a promising tool for early cancer screening.

### Construction of lung cancer detection models

**Fig. 5:**
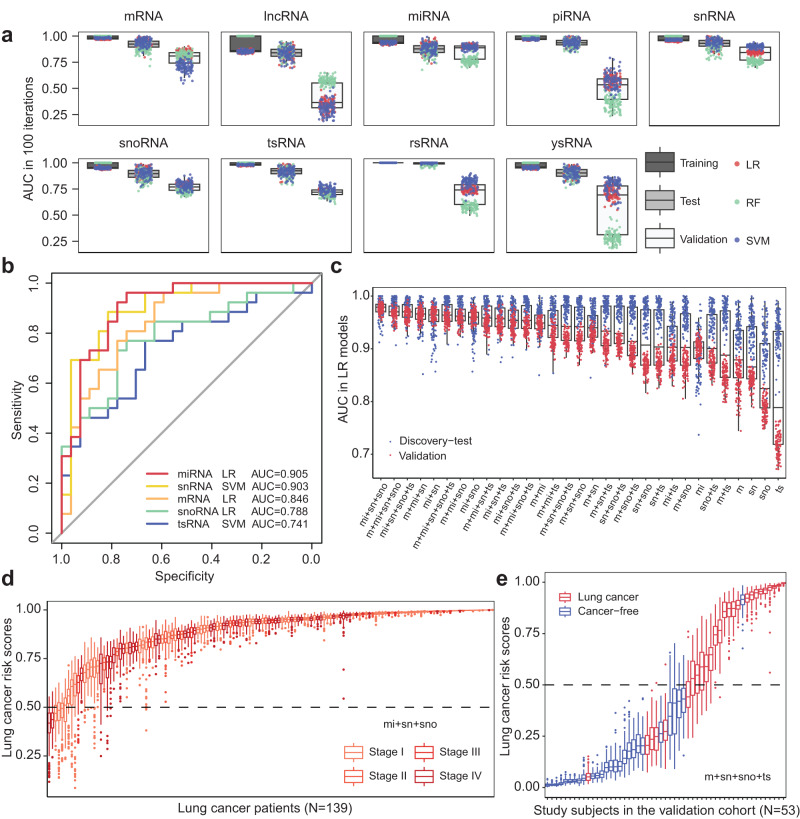
Evaluation of cfRNA signatures in early-stage lung cancer detection. **a** Area under the ROC curve (AUC) of nine different types of cfRNAs using three dissimilar machine learning classifiers (LR, RF, SVM) over 100 model iterations. The results in the 80% training set and 20% test set of the discovery cohort and the independent validation cohort (*N* = 26 cases and *N* = 27 controls) are shown. **b** ROC curves constructed from the median performance of 100 model iterations. The results of miRNA, snRNA, snoRNA, mRNA, and tsRNA in the validation cohort are shown. **c** AUC of Ridge regularized LR models constructed from different combinations of mRNAs, miRNAs, snRNAs, snoRNAs, and tsRNAs in the test set (blue) and the validation cohort (red). All models were repeated 100 times. **d** Lung cancer risk scores of different cancer stage (according to the AJCC/UICC 7th Edition) of LC patients predicted by 100 iterations of LR models trained with the “mi+sn+sno” panel. **e** Lung cancer risk scores of individual study subjects in the validation cohort predicted by 100 iterations of LR models trained with the “m+sn+sno+ts” panel. For all boxplots, the extremes of the boxes define the upper and lower quartiles, and the center lines define the median. Whiskers indicate 1.5× interquartile range (IQR). Source data are provided as a Source Data file.

Given that SLiPiR-seq successfully identified tumor-derived DE cfRNAs, we then investigated whether the utility of plasma transcriptome profiles could detect LC. We leveraged three different strategies to screen for candidate features: (1) filter method - “Top N”; (2) wrapper method - “Boruta”; (3) intrinsic method - “LASSO” (see Methods for details). To explore the potential of different kinds of RNAs in cancer detection, we carried out a series of machine-learning analyses. Three different algorithms (logistic regression (LR), random forest (RF), and support vector machine (SVM)) were applied to ensure the reliability of selected candidate features. We randomly partitioned the discovery cohort into 80% as a training set to train a diagnostic model and 20% as a test set to evaluate model performance. The sample partitioning and train-test processes were repeated 100 times for all feature selection and machine-learning analyses to avoid sampling bias. Consequently, we obtained 27 sets of candidate features, all of which can precisely detect LC in the training and test sets of the discovery cohort (Fig. 5a, Supplementary Table 1a, b). Since the candidate features identified by the LASSO method achieved the highest overall area under the receiver operating characteristic curve (AUC) in the test sets, we used the models trained with these features for subsequent validations.

To verify the performance of these models in detecting early-stage LC, we profiled an independent validation cohort of 26 stage I LC patients and 27 cancer-free individuals using SLiPiR-seq (Fig. 5a, Supplementary Table 1c). Among all candidate cfRNAs, miRNA features detected LC with the highest accuracy (LR AUC = 0.905 [interquartile range (IQR) 0.895–0.912]) (Fig. 5b). mRNA features also showed robust LC detection performance (LR AUC = 0.846 [0.823–0.860]). Interestingly, the seldom studied snRNA (SVM AUC = 0.903 [0.893–0.911]), snoRNA (LR AUC = 0.788 [0.772–0.798]), and tsRNA (SVM AUC = 0.741 [0.721–0.765]) features revealed considerably great ability in detecting LC as well (Fig. 5b). LR and SVM models training with rsRNA (SVM AUC = 0.819 [0.786–0.843]) or ysRNA (SVM AUC = 0.793 [0.747–0.829]) features showed high AUC in LC detection. However, models trained by the RF algorithm shared no agreement with these results (Supplementary Table 1c). Although the selected lncRNA and piRNA candidates demonstrated high AUC in the discovery cohort, none of them revealed LC detection capability in the validation cohort. Fifteen representative candidate cfRNAs showed concordant expression levels in both the discovery and validation cohorts (Supplementary Fig. 11). These observations indicate that SLiPiR-seq is promising in identifying not only commonly studied (miRNAs and mRNAs) but also rarely reported (snoRNAs, snRNAs, and tsRNAs) cfRNAs as cancer biomarkers.

### Combination of multiple types of cfRNAs achieved higher accuracy

Intrigued by the fact that cancer is a highly heterogeneous disease and that SLiPiR-seq has detected multifarious types of DE cfRNAs, we wondered if combining different RNA categories might improve model accuracy. To this end, we tested all possible combinations of the five types of candidate cfRNAs (aka. 29 mRNAs, 26 miRNAs, 10 snRNAs, 19 snoRNAs, and 38 tsRNAs, Supplementary Data 5) that have successfully undergone the ordeal of all three machine learning algorithms, resulting a total of 31 different combinations. We found that the combination of multiple RNA types provided more accurate results than using a single RNA type in most cases (Supplementary Fig. 12, Supplementary Data 6), suggesting that the combination strategy is beneficial for model performance.

To identify the best combination, we focused on the LR algorithm for calculating AUC, model sensitivity and specificity, and cancer risk scores in subsequent analyses. The top three most accurate combinations ranked by the median of AUC in the test set and validation cohort were “mi+sn+sno” (AUC = 0.979), “m+mi+sn+sno” (AUC = 0.970) and “mi+sn+sno+ts” (AUC = 0.970) (Fig. 5c). Next, we calculated the “risk scores” (aka. model-predicted probabilities) for all study subjects using the top panel “mi+sn+sno”. Of the 137 individuals in the discovery cohort who were assigned a high median risk score (≥0.5), 134 were true LC patients in the real world (97.81% specificity) (Supplementary Fig. 13a). Meanwhile, 134 of 139 LC patients in the discovery cohort were correctly diagnosed by this panel (96.40% sensitivity). As expected, we observed no evident differences in the model-predicted AUC and risk scores between early- and late-stage cancer patients (Fig. 5d, Supplementary Fig. 13b, c). In the validation cohort, all LC patients were diagnosed as high-risk individuals (100% sensitivity). However, the specificity of this panel is low in the validation cohort, 7 out of 33 individuals diagnosed as high-risk were not LC patients in the real world (78.79% specificity) (Supplementary Fig. 13d).

In the context of cancer screening, specificity often assumes a heightened importance than sensitivity. By ranking the specificities of all combinations (Supplementary Data 6), we identified an optimal panel for lung cancer screening in the general population. The “m+sn+sno+ts” panel shows 100% specificity and 99.28% sensitivity in the discovery cohort, and 95.24% specificity and 76.92% sensitivity in the validation cohort (Fig. 5e, Supplementary Fig. 13e). Prioritizing specificity can mitigate the multifaceted consequences of false-positive results, ensuring a more efficient and patient-centered approach to healthcare. These findings demonstrate the robust performance of cfRNA signatures in early-stage LC detection achieved by the integration of multiple types of cfRNAs.

### Establishment of cancer classification models

**Fig. 6:**
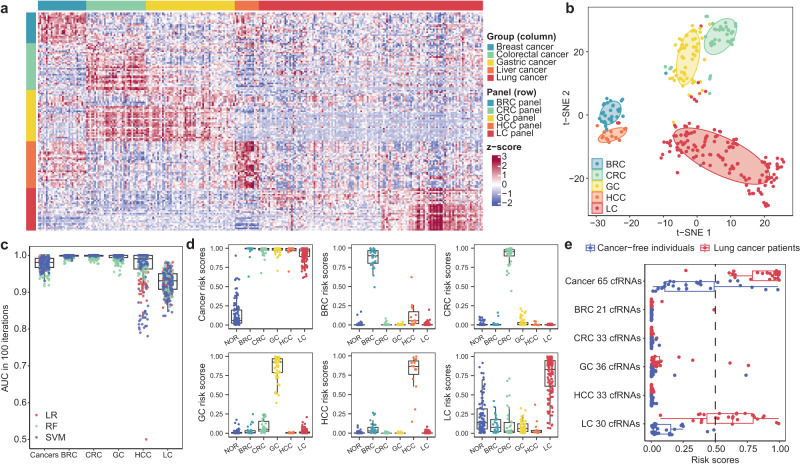
Utilization of cfRNA signatures for cancer classification. **a** Heatmap of the 21-cfRNA BRC panel, 33-cfRNA CRC panel, 36-cfRNA GC panel, 33-cfRNA HCC panel, and 30-cfRNA LC panel (row) between patients with different cancer types (column). Z score normalization was performed on the log_2_ transformed RPM normalized reads. **b** t-SNE plot showing clear separation of five different cancer types using cancer type-specific signatures (153 cfRNAs) selected by LASSO regularized LR. **c** AUC over 100 iterations of six sets of one-class versus-other-classes machine learning analyses using three different machine learning classifiers (LR, RF, SVM). **d** Median risk scores over 100 LR model iterations of all individuals in the discovery cohort and pan-cancer cohort predicted by a cancer detection model and five cancer type-specific detection models. *x* axis represents the actual disease state or tumor type of study subjects. **e** Median risk scores over 100 LR model iterations of individuals in the validation cohort predicted by different cancer-specific cfRNA panels. For all boxplots, the extremes of the boxes define the upper and lower quartiles, and the center lines define the median. Whiskers indicate 1.5× interquartile range (IQR). Source data are provided as a Source Data file.

To ascertain the clinical feasibility of SLiPiR-seq, we further explored its ability to identify cfRNA signatures for cancer classification. We expanded our study to encompass a pan-cancer patient cohort, comprising 30 breast cancer (BRC) patients, 37 colorectal cancer (CRC) patients, 55 gastric cancer (GC) patients, and 15 liver cancer (HCC) patients. The high Pearson correlation coefficients of the transcriptome profiles from distinct subject cohorts demonstrate that SLiPiR-seq can produce stable results under varying temporal and spatial conditions (Supplementary Table 2). We performed differential expression analyses for each cancer type in a one-class versus-other-classes paradigm (Supplementary Data 4), followed by LASSO LR to select discriminative features from the five RNA categories of interest (aka. mRNA, miRNA, snRNA, snoRNA, and tsRNA). Consequently, we identified five cancer type-specific panels (aka. 21-cfRNA BRC panel, 33-cfRNA CRC panel, 36-cfRNA GC panel, 33-cfRNA HCC panel, and 30-cfRNA LC panel) (Fig. 6a, Supplementary Fig. 14, Supplementary Data 5). Visualization using t-distributed stochastic neighbor embedding (t-SNE) revealed clear separation based on cancer type (Fig. 6b). We also combined all cancer patients as a group named “cancers” to compare with cancer-free donors and identified 65 cfRNAs that were commonly upregulated in all five cancer types studied. Subsequently, we conducted one-class versus-other-classes machine learning analyses on the six sets of candidates and observed high AUC values for the held-out test set samples across all classes (Fig. 6c, Supplementary Fig. 15).

We then assessed cancer risk scores as well as five cancer type-specific risk scores for all subjects. Notably, cancer-free individuals (NOR) exhibited low median risk scores in all six panels (Fig. 6d). All cancer patients exhibited high cancer risk scores in the 65-cfRNA common cancer panel. Furthermore, patients with a particular cancer type had high-risk scores only in the corresponding cancer type-specific panel but had low risk scores in the non-corresponding panels (Fig. 6d, Supplementary Fig. 16, Supplementary Data 7). The aforementioned independent validation cohort (*N* = 53) was used to verify the cancer classification performance of these panels. Remarkably, LC patients exhibited high median cancer risk scores (cancer 65 cfRNAs panel) and LC risk scores (LC 30 cfRNAs panel) while maintaining low risk scores in the BRC, CRC, GC, and HCC models (Fig. 6e). Cancer-free individuals had low median risk scores in all six models (Fig. 6e). Of the 27 cancer-free individuals, seven were erroneously assigned high cancer risk scores. Interestingly, only one was misclassified as GC but the remaining six cancer-free individuals had low risk scores in all five cancer type-specific models (Supplementary Data 7). This observation suggests that a two-step verification process could enhance the specificity of the cancer detection assay. A diagnosis should only be made when both the common cancer risk scores and at least one of the cancer type-specific risk scores are high. However, impaired sensitivity is expected as such a strategy will misclassify some real LC patients with only one positive risk scores. Collectively, these results demonstrate that cfRNA signatures identified by SLiPiR-seq are promising in the classification of different cancer types, motivating us to validate the clinical utility of SLiPiR-seq in larger sample cohorts in future investigations.

## Discussion

Compared to extensively investigated cfDNA as biomarkers for disease diagnosis and prognosis, cfRNAs are relatively poorly studied due to the absence of a robust and standardized methodology to comprehensively profile the highly fragmented cell-free transcriptome. Numerous studies have made efforts to optimize the library preparation protocol to analyze fragmented RNA with varied 5’ and 3’-ends^26,27,31,36,37^. However, none of these studies has systematically conducted case-control studies to validate the clinical feasibility of their optimized protocols. In this study, we not only developed a cfRNA profiling technology but also performed a comprehensive evaluation of nine different types of cfRNAs across the plasma transcriptome in cancer patients and cancer-free individuals.

This study began with benchmarking efforts to find out the optimal experimental and pre-analytical conditions for SLiPiR-seq, which will standardize the technology and set the stage for subsequent large-scale testing using clinical samples. To determine the accuracy of SLiPiR-seq in generating transcriptome profiles, we compared it with the gold standard RNA quantification method qPCR and the adapter ligation-based method NEBNext. The results of the comparisons demonstrated a high level of concordance among these techniques, indicating the robustness of SLiPiR-seq in capturing the RNA landscape. To our surprise, SLiPiR-seq identified significantly more unique cfRNA species and a broader range of fragment size than NEBNext, expanding the opportunity for the identification of candidate cfRNAs as disease biomarkers.

The use of splint ligation for small RNA library preparation has been reported in previous studies^31,38,39^. It is crucial to delineate the specific distinguishing features between SLiPiR-seq and the previous approach. Maguire et al. employed an RNA splint ligation adapter containing six degenerate nucleotides at the 5’ end of the RNA. This approach effectively increases ligation efficiency compared to traditional single-stranded RNA-RNA base adapter ligation, thereby increasing sensitivity, especially for low-input RNA libraries. However, even with this method, 5’-phosphate modification of the RNA remains a prerequisite for successful adapter ligation. In SLiPiR-seq, reverse transcription is performed prior to adapter ligation. Important sequencing elements, such as the sample barcode and the P7 primer sequences, are added to the first-strand cDNA after reverse transcription. SLiPiR-seq employs a DNA-based splint adapter that is capable of ligating to the 3’ end of the cDNA, corresponding to the 5’ end of the RNA. Consequently, this bypasses the necessity for a 5’-phosphate modification on the RNA.

The SLiPiR-seq technique has some limitations. First, a 3’-OH group is required for 3’-end polyadenylations. However, due to the random fragmentation characteristic of cfRNAs, a small fraction of them lacking a 3’-OH group cannot be analyzed by SLiPiR-seq. Second, modifications on RNA can disrupt the reverse transcription reaction by interfering with the Watson-Crick base pairing^40^. In the presence of a modification, the reverse transcription enzyme may dissociate from the RNA molecule, producing truncated reads. In the nine RNA categories investigated, tRNA molecules are extensively modified to fulfill their crucial role in decoding genetic information^41^. As expected, the majority of tsRNAs detected by SLiPiR-seq exhibit a fragment length of 18 nt because of the presence of the m^1^A modification at position 58 of tRNA, which is 18 nt away from the 3’-end^42^. Therefore, we encountered difficulties in accurately annotating tsRNAs due to the truncated reads. Despite this challenge, the potential value of tsRNA signatures for cancer detection and classification is evident. Third, circular RNAs in plasma are not identified by SLiPiR-seq as the design of this method is specifically sensitive to linear RNAs.

In our study, we employed three distinct machine learning algorithms (LR, RF, and SVM) to assess the candidate cfRNA signatures in the case-control studies. The convergence of results across different algorithms underscores the strength and validity of the identified biomarkers for their potential clinical application. Importantly, the results of mRNAs, miRNAs, snRNAs, snoRNAs, and tsRNAs obtained from these algorithms exhibited a high level of agreement, demonstrating the reliability of our findings. It is important to note that the accuracy achieved by the models in detecting early-stage lung cancer is likely due to the retrospective study design. Therefore, the presented model accuracy does not reflect the accuracy that would be achieved for lung cancer screening in the general population. However, the cfRNA signatures identified in our study may still hold significant value in real-world cancer screening.

Recent studies have reported that tsRNAs can regulate cancer progression at the post-transcriptional level through multiple mechanisms and are thus considered critical regulators and biomarkers of cancer^43–45^. When we sorted the average specificities of different cfRNA combinations in the discovery and validation cohort, we found that tsRNAs were present in all top five combinations (“m+sn+sno+ts”, “m+mi+ts”, “m+sn+ts”, “sn+ts”, “m+ts”) (Supplementary Data 6). This finding suggests that the inclusion of tsRNAs in the combination panel significantly enhances the specificity of the machine-learning models in lung cancer detection. This heightened specificity is of paramount importance in early cancer screening, where the cost and psychological implications of false positives can be substantial.

There are also some concerns regarding the clinical feasibility assessment of SLiPiR-seq. First, it is important to acknowledge that plasma samples of cases and controls were collected from different sites, and the age and sex were not rigorously matched due to the inherent challenges of sample collection. Therefore, the differences observed in transcriptome profiles between cases and controls may be influenced by confounding factors. However, it is worth mentioning that differences arising from confounding factors are unlikely to be consistently present across different cohorts. By applying our analysis to an independent validation cohort, we aimed to minimize the impact of such confounding factors and increase the reliability of our findings. Second, detailed clinical information, including risk factors such as the smoking history of cancer patients, was not systematically collected at the participating clinical centers. Consequently, we were unable to analyze the potential influence of these covariates on the plasma transcriptome profiles. Third, the small sample size of the pan-cancer patient cohort limited our ability to perform a comprehensive cancer classification test. Further studies with more cancer types and larger patient cohorts are needed to validate our findings.

In conclusion, we have developed a sensitive and robust approach for plasma transcriptome profiling that paves the way for comprehensive research on cfRNAs. We uncovered transcriptome landscapes of plasma from cancer patients and cancer-free individuals. Our findings require further validation in more independent cohorts, but this proof-of-principle study provides important insights into the potential future clinical applications of SLiPiR-seq to detect cfRNA signatures for sensitive and accurate early cancer detection and classification.

## Methods

### Sample acquisition and clinical cohorts

Pooled plasma samples of healthy individuals were used in the technology optimization part. In the clinical feasibility test part, study subjects were recruited from People’s Hospital of Bao’an Shenzhen (*N* = 111 cancer-free individuals, NOR_SZBA), The Fifth People’s Hospital of Suzhou (*N* = 27 cancer-free individuals, NOR_SZDW), The Second People’s Hospital of Shenzhen (*N* = 140 lung cancer patients, LC_SZDE), Peking University Shenzhen Hospital (*N* = 28 lung cancer patients, LC_SZBU), General Hospital of Ningxia Medical University (*N* = 30 breast cancer patients, BRC_NXYK; *N* = 37 colorectal cancer patients, CRC_NXYK; *N* = 55 gastric cancer patients, GC_NXYK; and *N* = 16 liver cancer patients, HCC_NXYK). Inclusion criteria for cancer patients were: (1) patient was diagnosed with cancer through histopathology; (2) patient has not undergone tumor resection surgery or any other form of therapy. The clinical characteristics of all study subjects are available in Supplementary Table 3. The study protocol was reviewed and approved by the Institutional Review Board of all participating sites. Informed written consent was obtained from each participant prior to sample collection.

### Whole blood processing

For all cohorts, 2 ml of whole blood samples were collected in EDTA anticoagulated vacutainers. Blood was drawn in the morning from participants who had fasted overnight. Plasma separation was done within 3 hours of whole blood collection. Blood samples were first centrifuged at 1600 × *g* for 10 min at 4 °C. The plasma layer was transferred to a clean tube and further centrifuged at 16,000 × *g* for 10 min at 4 °C to ensure complete depletion of cell debris. The double-spun plasma was split into 200 μl aliquots and stored at −80 °C until RNA isolation.

### cfRNA extraction

Frozen plasma samples were thawed on ice prior to cfRNA extraction. 200 μl of plasma samples were subjected to cfRNA extraction using the Apostle MiniMax^TM^ High-Efficiency cfRNA Isolation Kit (Apostle), following the manufacturer’s protocol with minor modifications. The experiments were conducted through manual operation on the bench up until the step involving the addition of 6.5 μl of Binding Enhancer to the 200 μl supernatant. The mixture was transferred to a 96-well plate with the prepared binding/nanoparticle solution, cfRNA Wash Solution, and 80% Ethanol (two columns) on different columns (eight samples in parallel). The subsequent steps, including binding and three rounds of magnetic nanoparticles washing, were performed using an automated magnetic bead handling device (TANBead Nucleic Acid Extraction System - Maelstrom 8 Autostage). After the final washing step, the liquid phase was transferred from the automation system to a new 1.5 ml tube, followed by the removal of any residual liquid in the tube and air drying on a magnetic rack. Lastly, cfRNA was eluted from the nanoparticles using 10 μl of RNase-free water. Due to the trace amounts of extracted cfRNA, it was directly employed for library preparation.

### SLiPiR-seq library preparation

Polyadenylations (poly A) and reverse transcription (RT) reactions were performed simultaneously in a 10 μl reaction system within 0.2 ml PCR tubes. This system consisted of 6.75 μl of cfRNA, 1.25 μl of 4× poly A/RT buffer^29^, 1 μl of 5 nM RT primer (GenScript Gene Synthesis, 5’-TACGAGATGTGACTGGAGTTCAGACGTGTGCTCTTCCGATCTNNNNNNNNTTTTTTTTTTTTTTTTTTVN-3’), and 1 μl of premixed poly-A polymerase (Enzymatics) and M-MLV reverse transcriptase (Fapon Biotech). The tube was incubated at 37 °C for 30 min in a thermocycler. The 8 nt of ‘NNNNNNNN’ in the RT primer sequence represent unique sample barcodes. To deplete any remaining RT primer, 1 μl of Exonuclease I (New England BioLabs) was added after the poly A/RT reaction, followed by incubation at 37 °C for 30 min and 80 °C for 20 min. Single-stranded cDNA was obtained through denaturation at 95 °C for 5 min.

The 11 μl RT reaction mix was then combined with a 9 μl master mix for splint ligation. The master mix comprised 1 μl of T4 DNA ligase (New England BioLabs), 1 μl of 10× T4 DNA Ligase buffer (New England BioLabs), 2 μl of 50% Polyethylene glycol 4000 (Thermo), 2 μl of 10 mM ATP (Enzymatics), 1 μl of 10 μM premixed synthetic adapter (GenScript Gene Synthesis, 5’-CACCTCTCTAUACACUCTTUCCCUACACGACGCTCTUCCGATCUNNNNNN-3’ and 5’-AGATCGGAAGAGCGTCGTGTAGGGAAAGAGTGTATAGAGAGGTG-3’), and 2 μl of double-distilled water. Splinted ligation occurred by incubating at 20 °C for 1 hour and 65 °C for 10 min. To digest the blocking strand of the adapter, 1 μl of USER enzyme (New England BioLabs) was added, followed by incubation at 37 °C for 15 min.

The libraries were amplified using 2× KAPA HiFi HotStart ReadyMix (Roche Diagnostics) in a 50 μl reaction system with synthetic forward primer (5’-AATGATACGGCGACCACCGAGATCTACACCTCTCTATACACTCTT-3’) and reverse primer (5’- CAAGCAGAAGACGGCATACGAGATGTGACTGGAGTT-3’). Amplification conditions were set as follows: activation at 95 °C for 3 min, followed by sixteen cycles of 98 °C for 20 s, 60 °C for 30 s and 72 °C for 30 s, and a final extension of 72 °C for 1 min. Amplified libraries were quantified by Qubit assay with the Equalbit 1× dsDNA HS Assay Kit (Vazyme). If the concentration of a sample was less than 10 ng/μl, additional amplification cycles were applied. The amplified libraries were purified using 1.8 times the volume of AMPure XP beads (Beckman Coulter), followed by quantification of the final libraries. To reduce RNA/DNA loss, the entire SLiPiR-seq library preparation workflow was performed within one tube. Libraries with different sample barcodes were pooled together and sequenced on the Illumina NovaSeq 6000 platform (HaploX, Shenzhen) to at least 10 million paired-end reads per sample.

### Raw reads processing

The adapter, sample index, and poly-A sequences at the 3’ end of R1 raw sequencing reads were cut away using Cutadapt^46^. Low-quality reads (*q* < 20) and reads less than 15 nt in length in the R1 file were trimmed away using Trimmomatic^47^. The first 8 nt of R2 raw sequencing reads (aka sample index), and their identities were extracted using awk. The trimmed R1 fastq files were then split to several subfiles based on the sample indices using a customized python script. The R1 subfiles were used for subsequent reads calling. The R2 files were discarded because their insert reads are of low quality due to the foregoing poly-T sequences. The data processing pipeline was accelerated by executing jobs in parallel in Linux shell using GNU Parallel^48^.

### Establishment of the reference genome and reads calling for tsRNAs, rsRNAs, and ysRNAs

Since there is no existing publicly available reference genome for rsRNAs and ysRNAs, the trimmed clean reads were aligned to the full-length Homo sapiens ribosomal RNAs (28 S, 18 S, 5.8 S, and 5 S rRNA) and Y RNAs (RNY1, RNY3, RNY4, and RNY5) sequences obtained from NCBI with zero mismatch tolerance (--score-min C,0) using Bowtie2^49^. Small RNA fragments mapped to rRNAs and Y RNAs were considered as rsRNA and ysRNA, respectively. Ordered ID were named to all unique rsRNAs and ysRNAs, and then their sequences were archived in a FASTA format file. The reference genome of tsRNA was obtained from MINTbase (https://cm.jefferson.edu/MINTbase/)^34^. Due to the high similarity of the sequences of parent tRNAs, alignment to a database of validated tsRNA sequences allows for more accurate quantification than alignment to a database of full-length tRNAs. The read counts of tsRNAs, rsRNAs, and ysRNAs were called using a custom python script that only counts when a fragment is 100% matched to one of the sequences in the FASTA file.

### Reads calling for miRNAs and piRNAs

Reference sequences of miRNAs and piRNAs were acquired from miRbase (https://www.mirbase.org/)^50^ and piRNABank (http://pirnabank.ibab.ac.in/)^51^, respectively. The clean reads were first mapped to these references using Bowtie2. Read counts of miRNAs and piRNAs were then called from the output SAM file using awk. Fragments less than 19 nt in length were further trimmed away before miRNA read calling to avoid misclassifications.

### Reads calling for lncRNAs, mRNAs, snRNAs, and snoRNAs

The clean reads were mapped to the GRCh38 (hg38) reference genome using Bowtie2. The expression level of mRNAs, lncRNAs, snRNAs, and snoRNAs were called with the GENCODE annotation (release 41, https://www.gencodegenes.org/human/release_41.html) using featureCounts^52^ from the Subread package. Additional trimming of fragments less than a length of 23 nt was performed before mRNA and lncRNA read calling to reduce the multiple alignment rate.

### Quality controls of sequencing results

The called read counts of each sample were merged into an expression matrix using R, followed by reads per million (RPM) normalization and log transformation. The number of total reads, clean reads (≥15 nt), and mappable reads (mapped to GRCh38) were calculated. The sum of reads for each of the nine RNA types, and their percentage ratios were calculated. The number of RNA species detected (RPM > 0) for each RNA type was also calculated. Exclusion criteria of low-quality samples were: (1) Clean reads ratio <20%; (2) Clean reads <2 million; (3) rsRNA ratio > 30%; (4) the sum of lncRNA and mRNA ratio > 30%. 9 out of 444 samples were excluded based on these criteria. The reproducibility of SLiPiR-seq was measured by transcriptome-wide Pearson correlation using the normalized read counts of each detected cfRNA.

### Technology optimizations

Plasma samples from multiple healthy donors were pooled together for the optimizations of SLiPiR-seq. The SLiPiR-seq library construction was carried out using either the full protocol described above, or a modified protocol with a single variable, including the replacement of ExoI or USER enzyme with distilled water, different final concentrations (1.25 nM, 2.5 nM, 5 nM, 10 nM, 20 nM, 40 nM, and 80 nM) of RT primers, different input plasma volumes (12.5 μl, 25 μl, 50 μl, 100 μl, 200 μl, and 400 μl), different blood standing time before plasma separation (3 h, 6 h and 9 h), and different freeze-thaw cycles of plasma (FT0, FT1, FT2, and FT3). The same number of total reads was randomly subsampled from the sequencing results of different conditions for subsequent reads calling.

### Quantitative real-time PCR

Single-stranded cDNA was obtained through the same procedures as described in the SLiPiR-seq library preparation section. qPCR was performed based on the S-Poly(T) Plus method: 1 μl of cDNA template (1:25, 1:10, 1:10, 1:20, 1:2, 1:20, 1:2, 1:20, and 1:10 dilution of original cDNA for lncRNA, miRNA, piRNA, mRNA, snRNA, rsRNA, snoRNA, ysRNA, and tsRNA, respectively), 0.2 μl of AceTaq DNA Polymerase (Vazyme), 2 μl of 10× AceTaq Buffer (Vazyme), 0.4 μl of 10 mM dNTP Mix (Vazyme), 0.2 μl of 100× ROX (Sigma-Aldrich), 0.5 μl of 5× SYBR Green (Roche Diagnostics), 5 μl of 1 μM universal reverse primer (5’-TACGAGATGTGACTGGAGTT-3’), 5 μl of small RNA specific forward primer (Supplementary Data 1), and add double-distilled water up to 20 μl. Amplification conditions were set as follows: activation at 95 °C for 5 min, followed by forty cycles of 95 °C for 10 s, 60 °C for 30 s. Assays were performed on ABI StepOne plus a real-time PCR system (Applied Biosystems).

### 5’-phosphate modification detection tests

RNA oligos synthesized with or without 5’-phosphate modification (Supplementary Data 2) (GenScript Gene Synthesis) were resuspended at a concentration of 100 μM in TE buffer. Equimolar of RNA oligo was mixed at a final concentration of 10 μM. 1 μl of the mixture was used for library preparation using NEBNext Small RNA Library Prep Set (New England Biolabs) according to the manufacturer’s protocol and SLiPiR-seq as described above.

### Calculation of coverage and fragment length distribution

cfRNA was extracted from the pooled plasma for the comparison of NEBNext and SLiPiR-seq. 20 million clean reads were randomly subsampled from the sequencing results of both methods and were used for subsequent reads calling. Fragment length distributions were calculated based on the size and depth of sense-aligned reads. Exon and intron coordinates were extracted from mRNA and lncRNA annotations in GENCODE (release 41). Aligned reads obtained from sam files were used to determine “sense” or “antisense” in exon and intron regions. Gene body coverage of both NEBNext and SLiPiR-seq was evaluated using the ‘geneBody_coverage.py’ function in the RSeQC package with corresponding bam files and GRCh38 reference genome^53^.

### Cross-analysis of tissue-specific genes

Tissue-specific genes were obtained from the Human Protein Atlas database (https://www.proteinatlas.org/humanproteome/tissue) by selecting the ‘The Tissue-specific Proteome’ in the ‘Tissue’ tag. 131 breast, 919 intestine, 441 kidney, 956 liver, 127 lung, 145 ovary, 303 pancreas, 127 prostate, and 307 stomach tissue elevated genes were selected to intersect with mRNAs detected by NEBNext and SLiPiR-seq (Supplementary Data 2).

### Sample grouping and partitioning

In the lung cancer study, samples from NOR_SZBA and LC_SZDE were grouped as a discovery cohort (*N* = 245). In the pan-cancer study, samples from NOR_SZBA, LC_SZDE, BRC_NXYK, CRC_NXYK, GC_NXYK and HCC_NXYK were grouped as a discovery cohort (*N* = 382). Samples from NOR_SZDW and LC_SZBU were grouped as a validation cohort (*N* = 53) to validate models established in both the lung cancer detection study and the pan-cancer classification study. The discovery cohort was randomly partitioned into training and test sets in an 80–20% manner using the createDataPartition function in the R package caret^54^. The training set was used to train models, and the held-out test set was used to assess the models. The independent validation cohort represents unbiased measures of model accuracy, as samples in this cohort were not used for either candidate feature selection or model training and tuning.

### Differential expression analyses

The raw count matrices were fitted to a negative binomial distribution using the R package DESeq2^55^. DE cfRNAs were identified at a significance level of 0.1 Benjamini–Hochberg false discovery rate (BH-FDR, Wald test). Differential expression analyses were implemented on (1) SLiPiR-seq cfRNA data of lung cancer patients versus cancer-free controls in the discovery cohort; (2) SliPiR-seq cfRNA data of both the discovery and pan-cancer cohorts in a one-class versus-other-classes paradigm. Volcano plots were made by the EnhancedVolcano R package. Pie chart was used to visualize the distribution of different RNA types in the DE cfRNAs. Inclusion criteria for initial candidates are: (1) Upregulated in cancer (fold change > 0); (2) moderate expression level (mean read counts across all samples >10); (3) large differences between cases and controls (log_2_ fold change >0.8).

### Candidate feature selection

Three different strategies were applied to select candidate features for lung cancer detection: (1) Filter method—‘Top N’, based on the rank of significance level; (2) Wrapper method—‘Boruta’, a relevant feature selection wrapper algorithm; (3) Intrinsic method—‘LASSO’, based on least absolute shrinkage and selection operator (LASSO) regularized binomial generalized linear model (GLM). All strategies were applied to each type of cfRNA individually.

In the self-developed filter method, different combinations of the top *N* cfRNAs ranked by significance level in the differential expression test were examined. Candidate features were selected by first combining the most and the second most significant cfRNAs (*N* = 2), and then sequentially adding the next significant one after another. For example, *N* = 3 means the combinations of top three significant cfRNAs. The best value of N was determined by the mean Area Under the receiver operating characteristic Curve (AUC) in the test set across three machine learning algorithms (see the next section for details). Features that negatively affected the mean testing set AUC were further filtered out. For example, if AUC of the top three cfRNAs was smaller than AUC of the top two cfRNAs, the third most significant cfRNA was removed from the combinations. The remaining features were verified in the independent validation cohort.

In the wrapper method, Boruta algorithm was applied using the R package Boruta. The variable importance measure was based on RF. The maximal number of important source runs was set to 500. The importance measurement process was repeated 100 times in the randomly partitioned training set. Features evaluated as high importance at least 10 times of the 100 repeats were considered as candidates for verification in the held-out test set and validation cohort.

In the intrinsic method, LASSO regularization of binomial GLM was constructed using the R package glmnet^56^. ‘Lambda.1se’ determined by 10-fold cross-validation was used as it gives a less complicated model while maintaining a low deviance. LASSO regression coefficient of each cfRNA was calculated for 100 times in the randomly partitioned training set. Features evaluated with a non-zero coefficient at least 10 times of the 100 repeats were considered as candidates. The candidates were further verified in the test set and validation cohort. In the cancer classification analysis, ‘LASSO’ method was used to select candidate features from the six sets of one-class versus-other-classes DE cfRNAs. Over 100 iterations, features evaluated with a non-zero coefficient at least 10 times were considered as candidates.

### Machine learning analyses

Three different machine learning algorithms, including logistic regression (LR), random forest (RF), and support vector machine (SVM), were applied in this study using the R packages glmnet, randomForest^57^, and caret, respectively. For the selection of candidate features, LASSO regularized (alpha = 1) LR was used. For the verification of selected candidate features, Ridge regularized (alpha = 0) LR was used. 10-fold cross-validation was done to determine the minimum lambda penalty value in all LR models. Random forest models were grown by 500 decision trees with default parameters. Likewise, Support Vector Machines with Linear Kernel (‘svmLinear’) was done using default parameters. All sample partitions and model train-test processes were repeated 100 times to prevent random sampling biases. The performance of models was evaluated by AUC and predicted risk scores of individuals. Receiver operating characteristic (ROC) curve was plotted by the R package pROC. AUC was calculated by the R package ROCR. Risk score was the probability of individual sample predicted by the model. Sensitivity was the ratio of LC patients with a high median risk score to all LC patients. Specificity was the ratio of LC patients who received a high median risk score to all individuals with a high median risk score.

### Statistics and reproducibility

All experiments for the technology optimizations of SLiPiR-seq were repeated at least three times to ensure reproducibility. All sample partitions and model training and testing processes were repeated 100 times to avoid random sampling biases. Unless otherwise stated, comparison of means was performed with a two-sided, unpaired Welch’s t-test. Differential expression analyses were performed with Wald test and multiple testing correction (BH-FDR). No statistical method was used to predetermine the sample size. Nine out of 444 samples were excluded due to low sequencing data quality (see the exclusion criteria above). Samples were randomized and blinded during cfRNA extraction, library preparation, and sequencing. Samples were unblinded during data processing and analysis.

### Reporting summary

Further information on research design is available in the Nature Portfolio Reporting Summary linked to this article.

### Supplementary information

Supplementary Information

Peer Review File

Description of Additional Supplementary Files

Dataset 1

Dataset 2

Dataset 3

Dataset 4

Dataset 5

Dataset 6

Dataset 7

Dataset 8

Dataset 9

Reporting Summary

### Source data

Source Data

## Data Availability

Raw sequencing data (fastq) and raw read count matrix for technology optimizations are available through the Sequence Read Archive [SRA, PRJNA962827] and Supplementary Data 8, respectively. Raw read count matrices for all detected cfRNAs and all de-identified studied clinical samples are available in Supplementary Data 9. The raw sequencing data for clinical samples reported in this paper have been deposited in the Genome Sequence Archive^58^ in National Genomics Data Center^59^, China National Center for Bioinformation/Beijing institute of Genomics, Chinese Academy of Sciences [GSA-Human: HRA004959]. The raw sequencing data are under controlled access and are available upon request from the corresponding author to comply with the ethics regulation of Shenzhen University. Applicants should have obtained ethical approvals from their ethics committees and submitted a research proposal for the data request. Timescale for access to be granted would be around one month, and there are no restrictions on the duration of access. Reference genome: miRNA (miRbase, https://www.mirbase.org/), piRNA (piRNABank, http://pirnabank.ibab.ac.in/), tsRNA (MINTbase, https://cm.jefferson.edu/MINTbase/), GRCh38 (hg38, https://hgdownload.soe.ucsc.edu/goldenPath/hg38/bigZips/), GENCODE (https://www.gencodegenes.org/human/stats_41.html). Source data are provided in this paper.

## Code availability

All computational analyses were performed using Linux shell, Python 3, or R 4.2. Scripts used to generate the findings in this study are available on Github at https://github.com/JinyongHuang/SLiPiR-seq (10.5281/zenodo.10128082).
