# Supplementary material for: Terminal modifications independent cell-free RNA sequencing enables sensitive early cancer detection and classification
Source: Nat Commun. 2024 Jan 2;15:156. doi: 10.1038/s41467-023-44461-y (PMC10761679; doi:10.1038/s41467-023-44461-y)
Supplement: Supplementary file 1 — Supplementary Information [file 41467_2023_44461_MOESM1_ESM.pdf]

Supplementary Figures

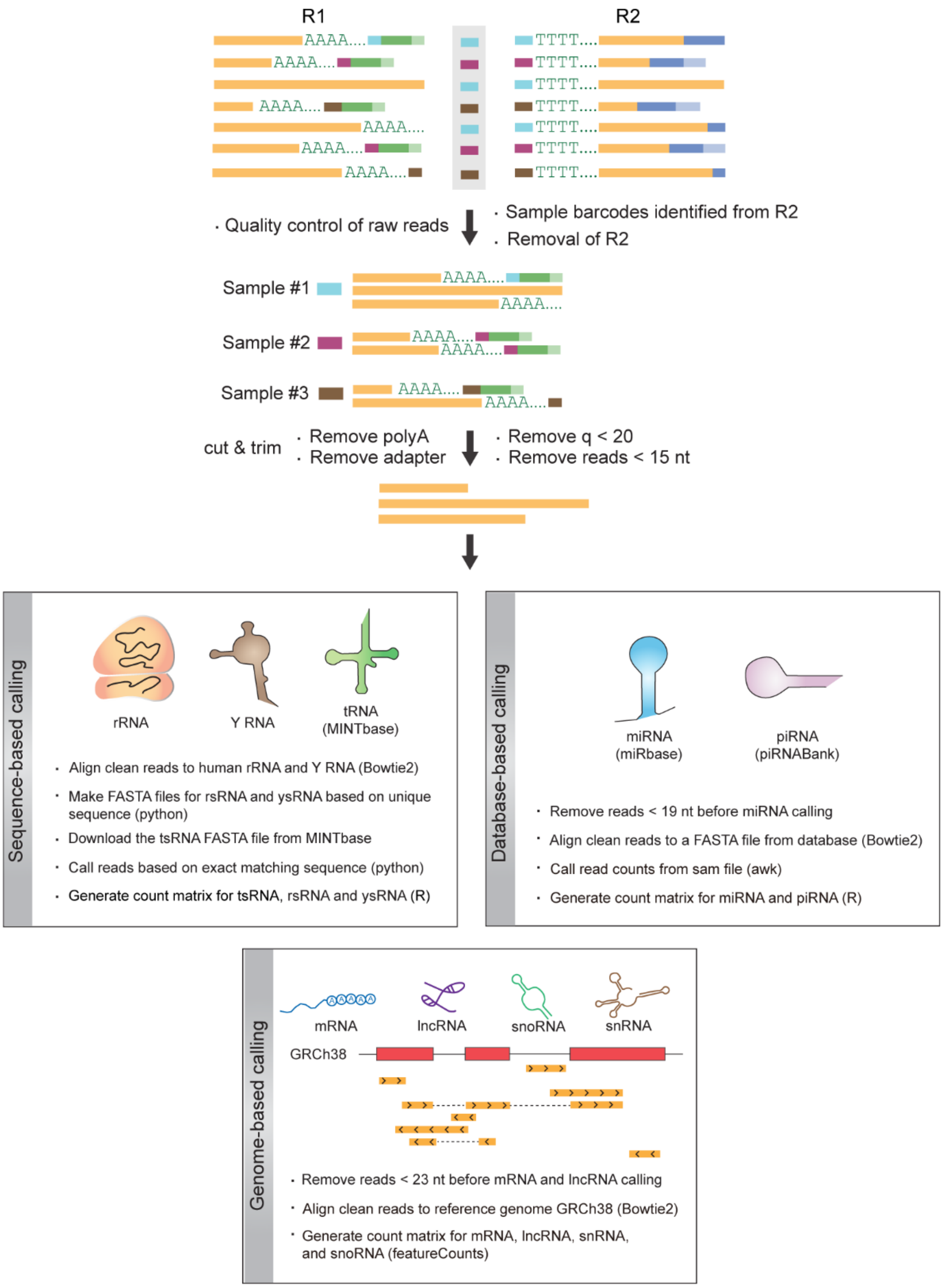

Supplementary Fig. 1 | Schematic diagram of the bioinformatics data processing workflow for reads calling of nine different RNA types. R1 and R2 indicate the forward reads and reverse reads from paired-end sequencing, respectively.

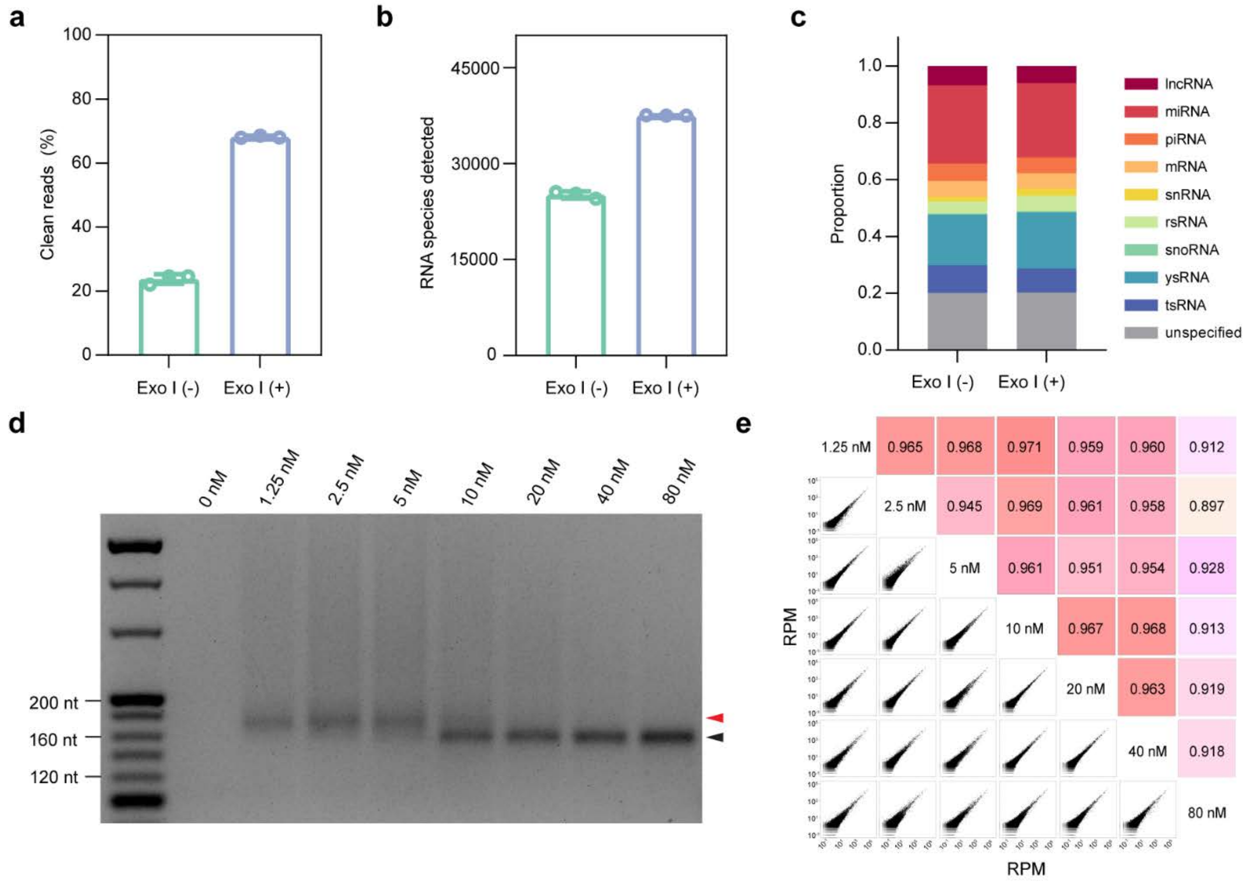

Supplementary Fig. 2 | a. Clean read ratio (Mean and SD) of the library prepared with or without Exo I treatment. **b.** The total number of cfRNA species detected (RPM>0, Mean and SD) from the library prepared with or without Exo I treatment. **c.** The proportion of different kinds of RNAs (Mean) in the library prepared with or without Exo I treatment. **d.** Gel electrophoresis showing the effect of different concentration of RT primer (from 1.25 nM to 80 nM). Black arrow indicates the size of the ligation product of RT primer and adapter. Red arrow indicates the correct cfRNA library size. **e.** Transcriptome wide Pearson's correlation of normalized reads (Mean) of all detected cfRNAs between different concentration of RT primer. For all graphs, N=3 technical replicates were performed for all conditions.

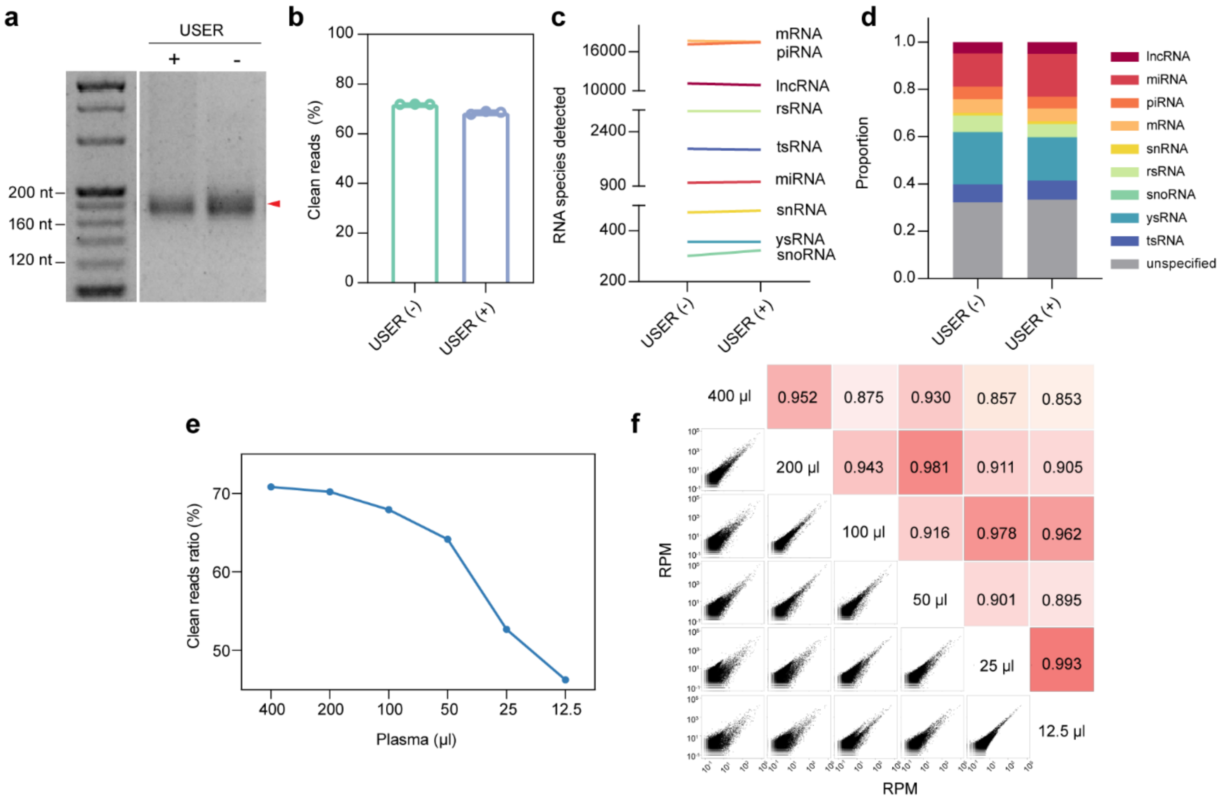

Supplementary Fig. 3 | a. Gel electrophoresis showing the effect of USER enzyme treatment. Red arrow indicates the correct cfRNA library size. **b-e.** Clean read ratio (Mean and SD) (b), the number of cfRNA species detected (RPM>0, Mean) for nine RNA types (c), and the proportion of different kinds of RNAs (Mean) (d) from the library prepared with or without USER enzyme treatment. **e.** Clean read ratio (Mean and SD) of the libraries constructed from different starting volumes of plasma. **f.** Transcriptome wide Pearson's correlation of RPM normalized reads (Mean) of all detected cfRNAs between different starting volumes of plasma. For all graphs, N=3 technical replicates were performed for all conditions.

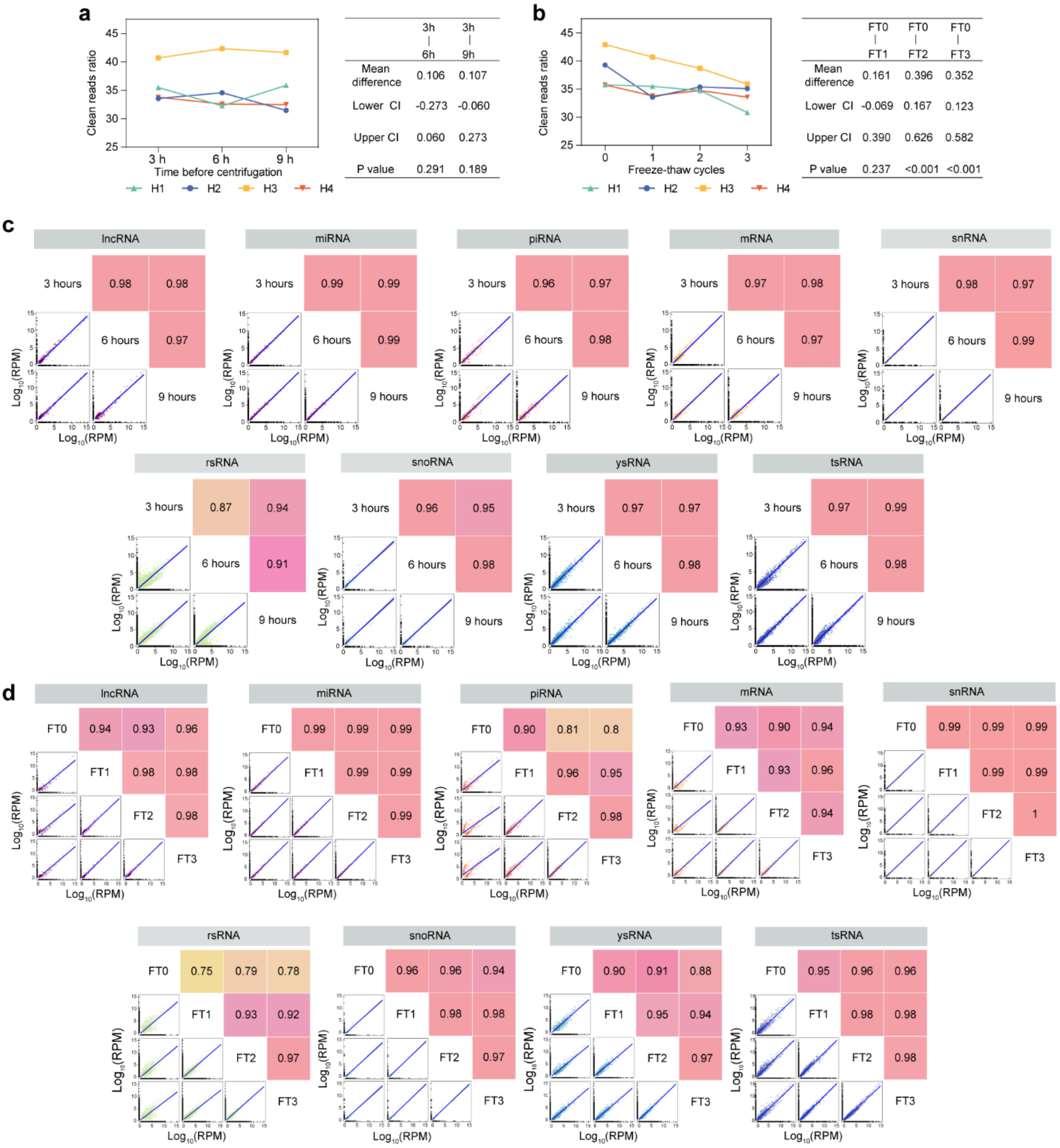

Supplementary Fig. 4 | a & b. Effect of blood standing time before plasma separation (3, 6 and 9 hours) (a) and effect of freeze/thaw cycles of plasma (FT0-3) (b) on the clean read ratio (Mean) of sequencing data (N=4 biological replicates). P values were determined using Tukey's test (a) and Dunnett’s test (b). **c & d.** Pearson’s correlation of normalized reads (Mean) of all nine different RNA types from libraries prepared with different blood standing time before plasma separation (c) and different freeze/thaw cycles of plasma (d) (N=4 biological replicates).

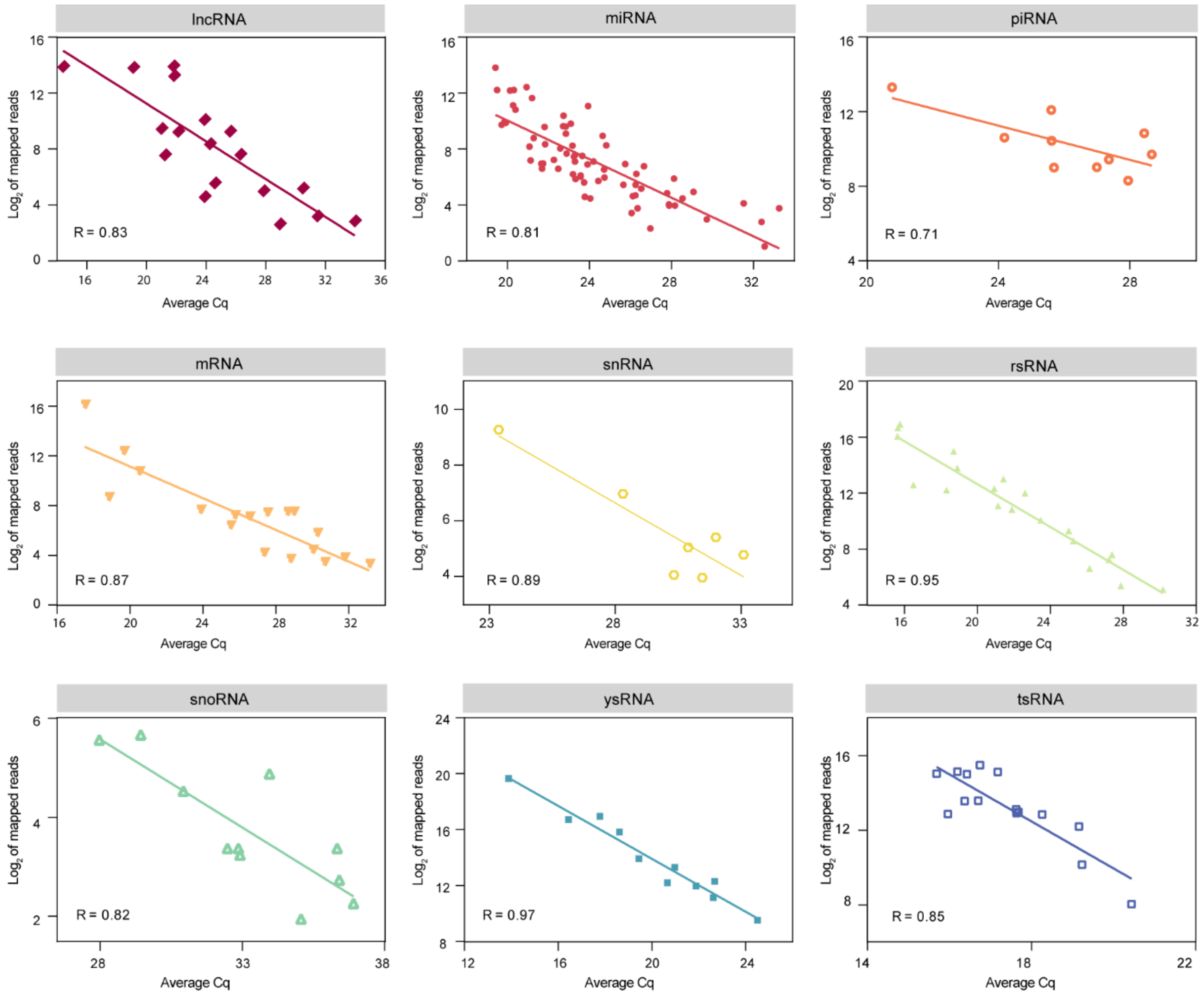

Supplementary Fig. 5 | Linear models fitted to log_2 RPM normalized sequencing reads profiled by SLiPiR-seq and Ct value (Mean, N=2 technical replicates) measured by qPCR. The Pearson correlation coefficient (R) values are shown on the bottom left. The results of nine different RNA types are shown.

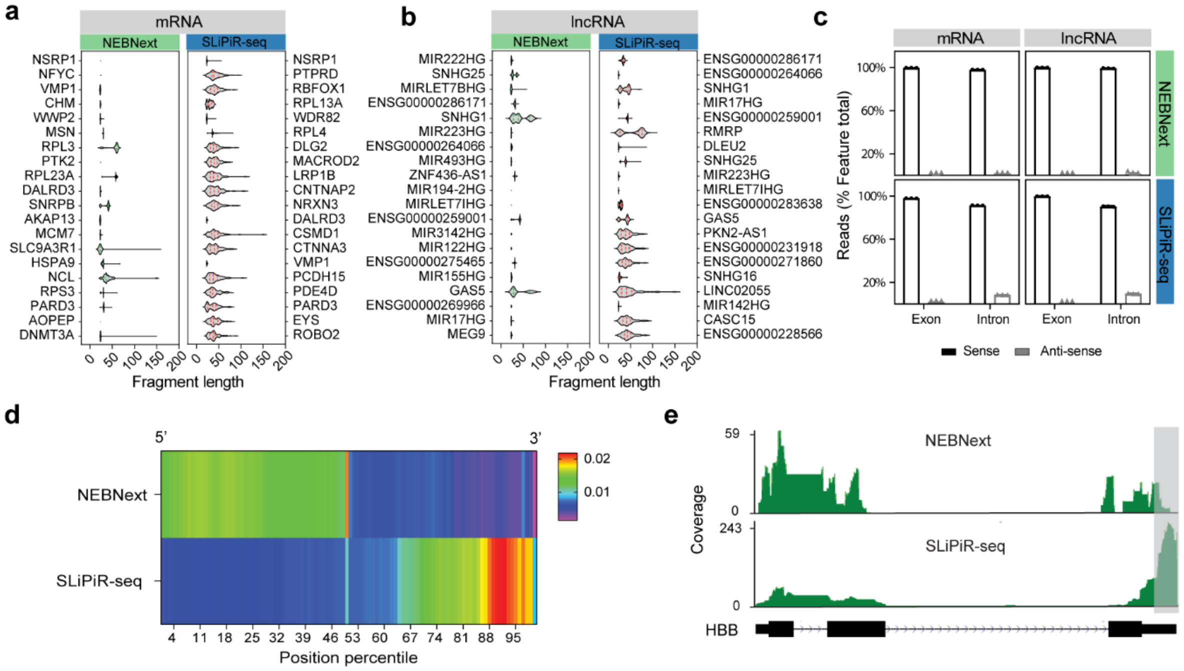

Supplementary Fig. 6 | a & b. Violin plots illustrating the fragment length distributions for the twenty most highly abundant mRNAs (a) and lncRNAs (b) across seven healthy donors in NEBNext and SLiPiR-seq. **c.** Bar plots showing the percentage of uniquely mapped reads from the sense (black) and antisense (grey) strands annotated to mRNA and lncRNA exons and introns measured by both methods. Mean and SD of percentages in three technical replicates are shown. **d.** Gene body coverage by relative position percentile from 5' end to 3' end of all annotated reads in both methods. SLiPiR-seq reads were mainly distributed at the 3' end of genes. **e.** Read coverage for the Hemoglobin (HBB) transcript in the libraries generated by the two methods as an example. Gray rectangle indicates the enrichment of sequencing reads at the 3' end of SLiPiR-seq results.

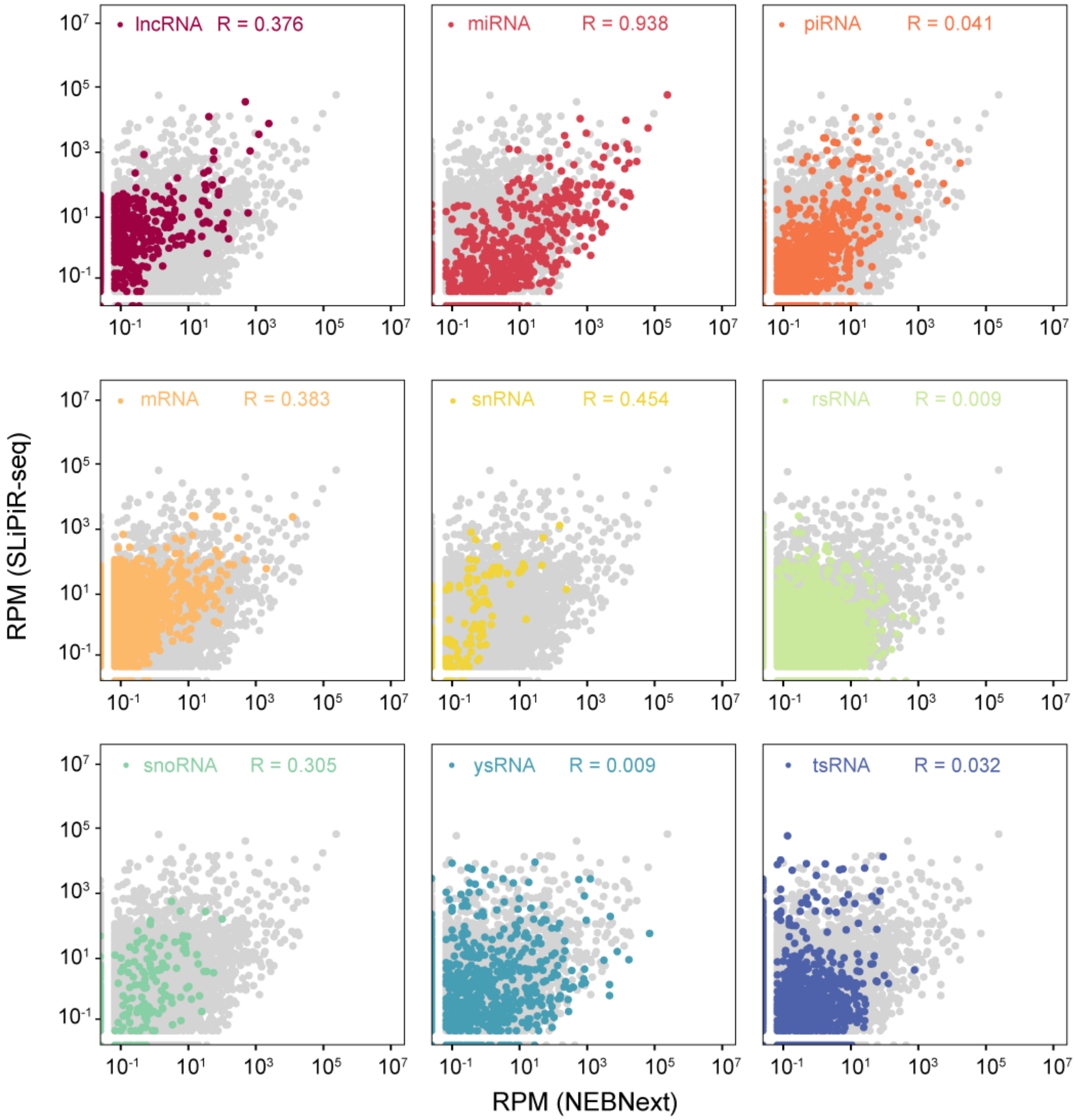

Supplementary Fig. 7 | Scatter plots showing the $\log_{10}$ transformed and RPM normalized read count (Mean, N=3 technical replicates) of RNA species detected by NEBNext and SLiPiR-seq. The Pearson correlation coefficient (R) values are calculated.

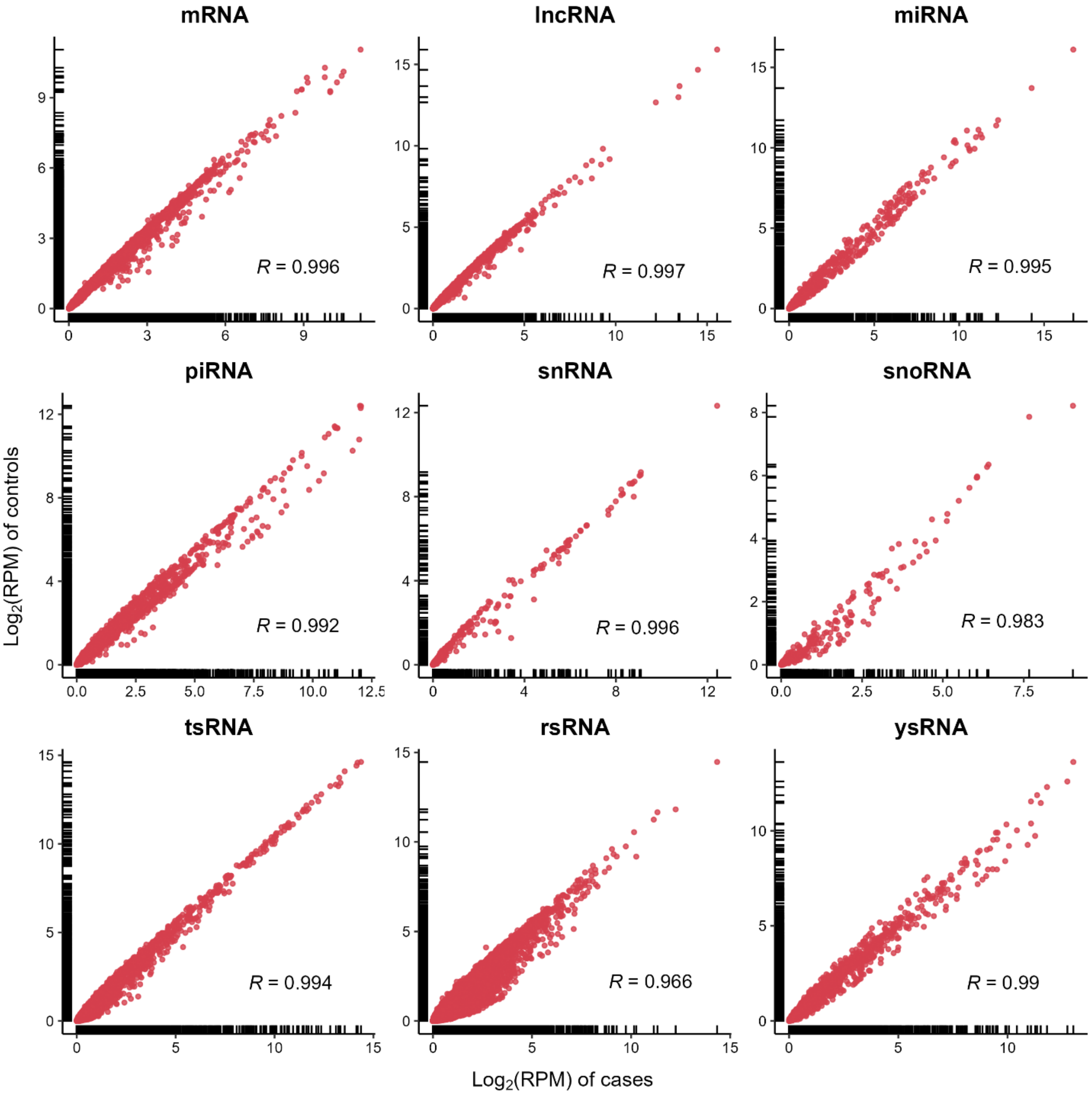

Supplementary Fig. 8 | Transcriptome wide Pearson's correlation of $\log_2$ transformed RPM normalized reads of different types of detected cfRNAs between cases (N=139) and controls (N=106).

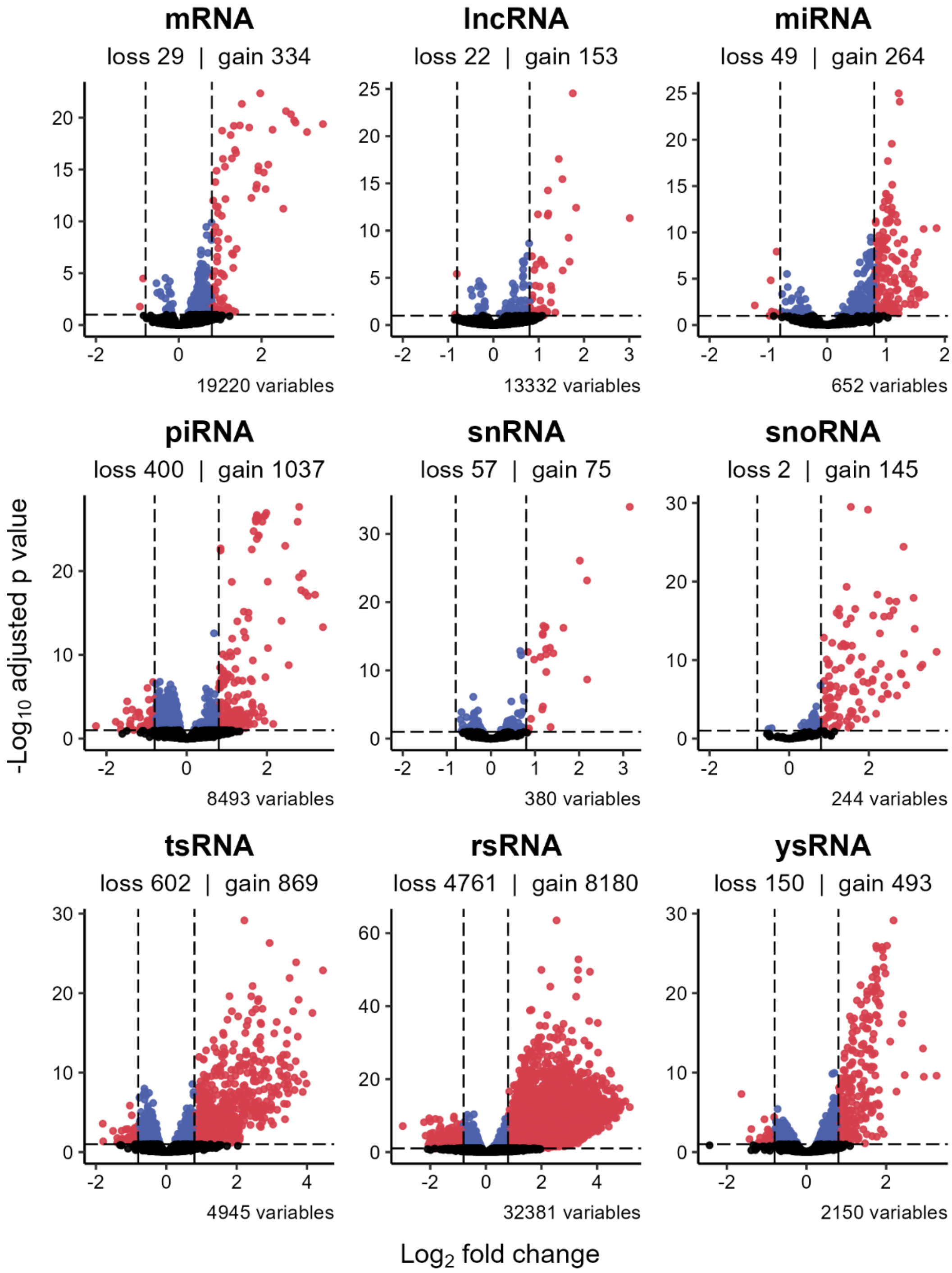

Supplementary Fig. 9 | Volcano plot of different types of DE cfRNAs from LC patients (cases, N=139) versus cancer-free donors (controls, N=106). Red dots indicate cfRNAs at BH-FDR < 0.1 (negative binomial GLM, Wald test, two-sided P values) and absolute log_2 fold change > 0.8.

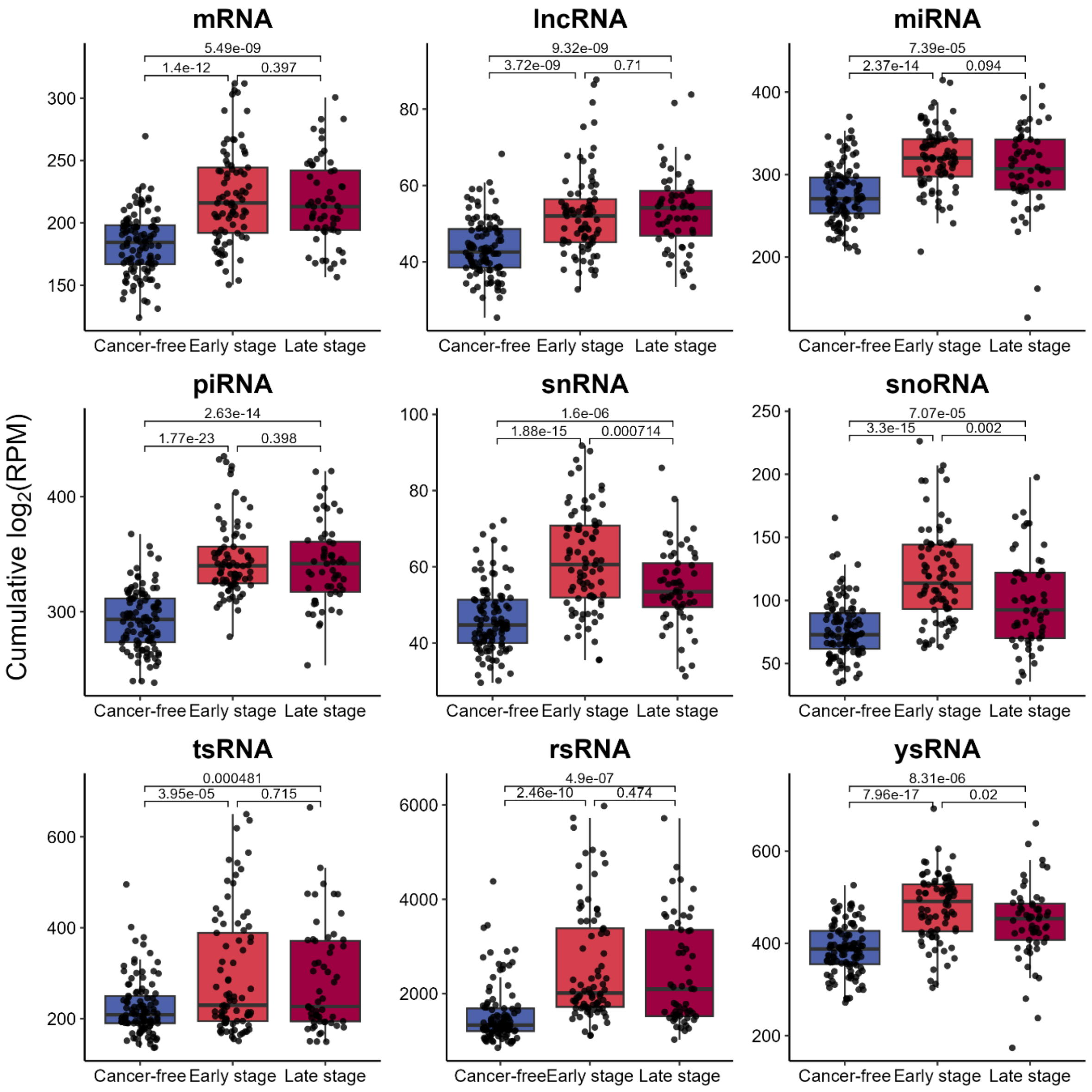

Supplementary Fig. 10 | Cumulative sum of the $\log_2$ transformed RPM normalized reads of different types of upregulated (fold change >0) DE cfRNAs between early-stage LC patients (N=81), late-stage LC patients (N=57) and controls (N=106) (Welch's t-test, two-sided P values).

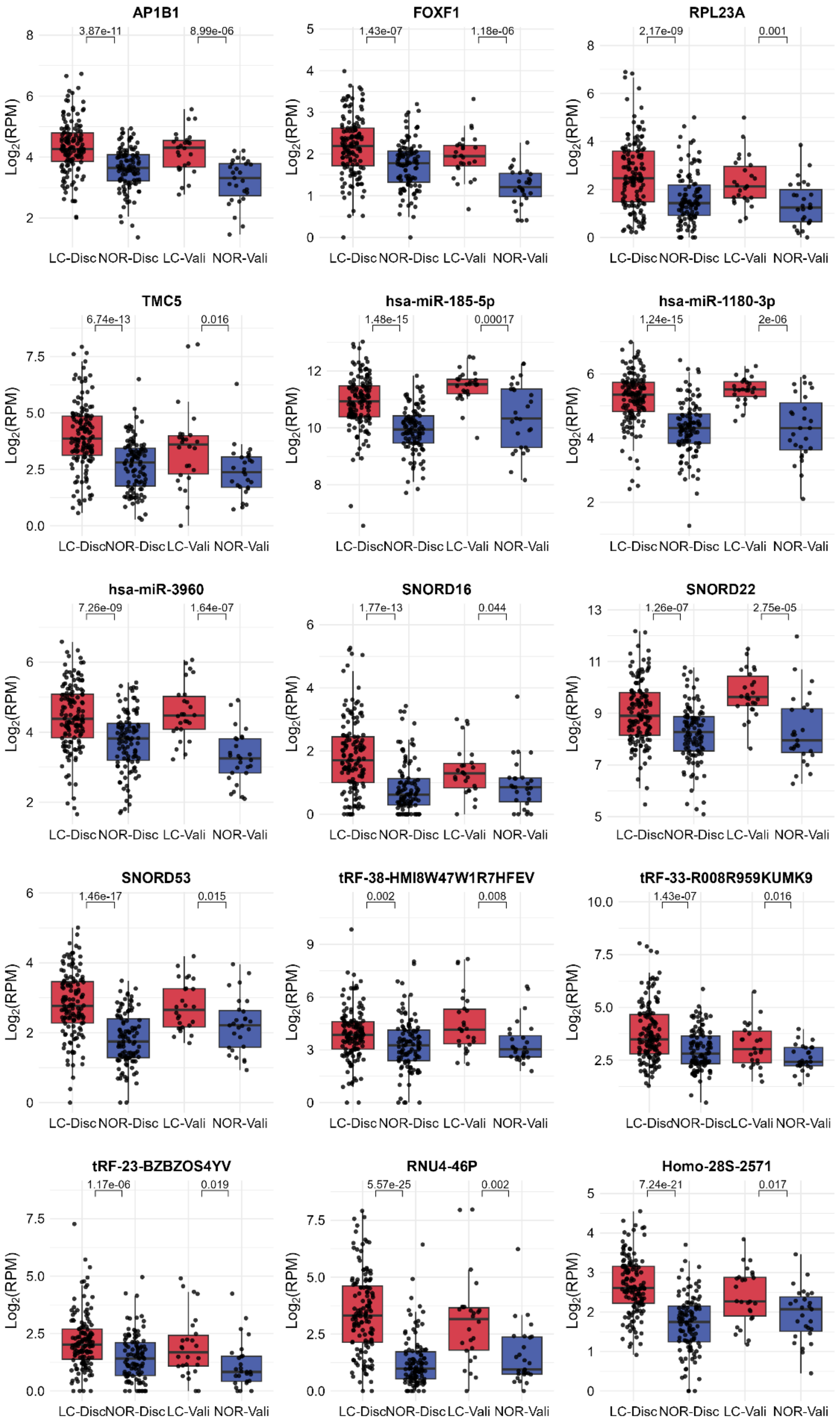

Supplementary Fig. 11 | The $\log_2$ transformed RPM normalized reads between cases and controls in the discovery and validation cohorts of fifteen representative candidate cfRNAs, including mRNAs (*AP1B1*, *FOXF1*, *RPL23A*, and *TMC5*), miRNAs (*hsa-miR-185-5p*, *hsa-miR-1180-3p* and *hsa-miR-3960*), snoRNAs (*SNORD16*, *SNORD22* and *SNORD53*), tsRNAs (*tRF-38-HMI8W47W1R7HFEV*, *tRF-33-R008R959KUMK9* and *tRF-23-BZBZOS4YV*), snRNA (*RNU4-46P*), and rsRNA (*Homo-28S-2571*, GGGGCCGGGGGTGGGGT) (Welch's t-test, two-sided P values). The extremes of boxes define the upper and lower quartiles, and the center lines define the median. Whiskers indicate 1.5× interquartile range (IQR).

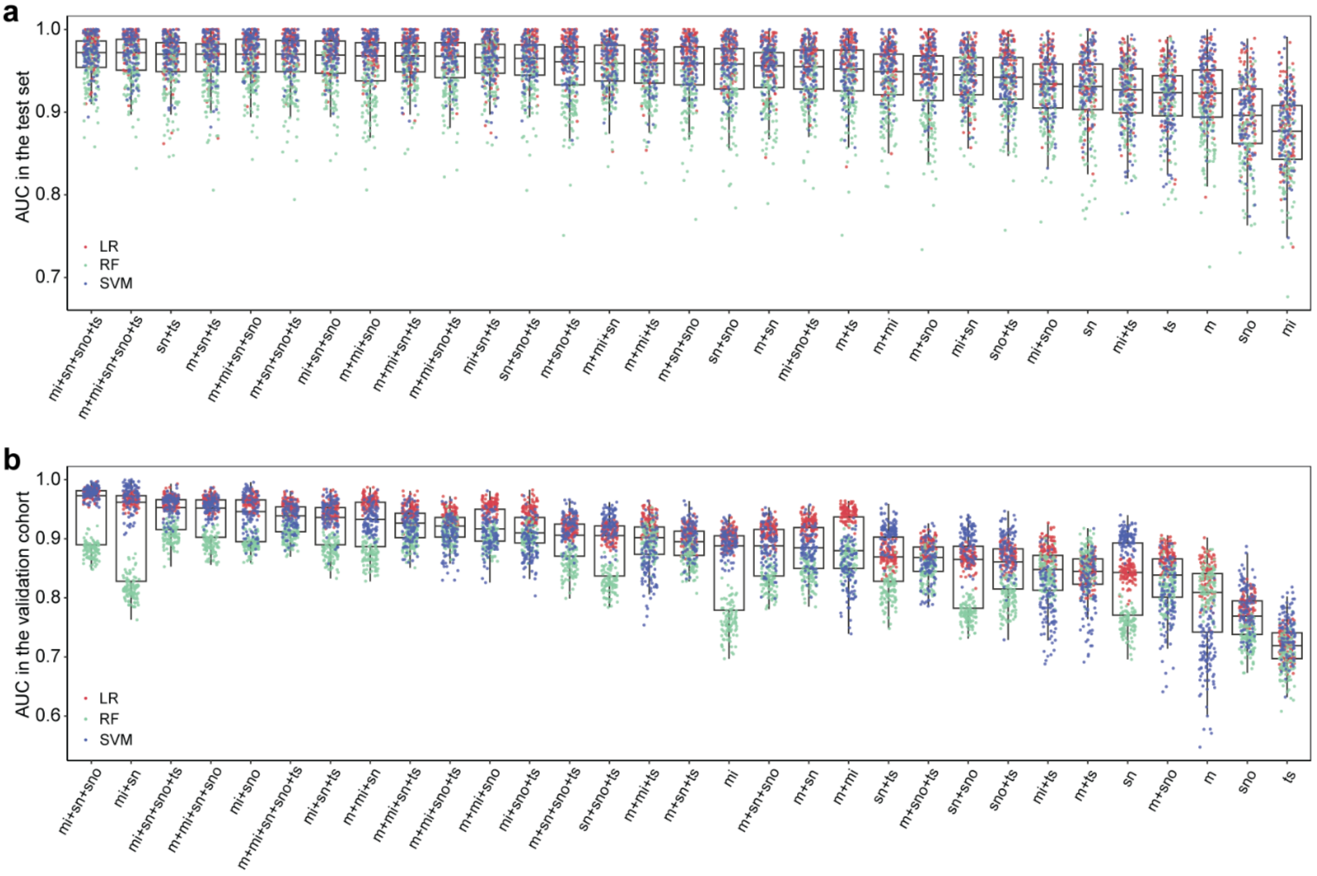

Supplementary Fig. 12 | Median AUC over 100 model iterations of three classifiers of different combinations of cfRNAs in the discovery cohort and validation cohort. The extremes of boxes define the upper and lower quartiles, and the center lines define the median. Whiskers indicate 1.5× interquartile range (IQR).

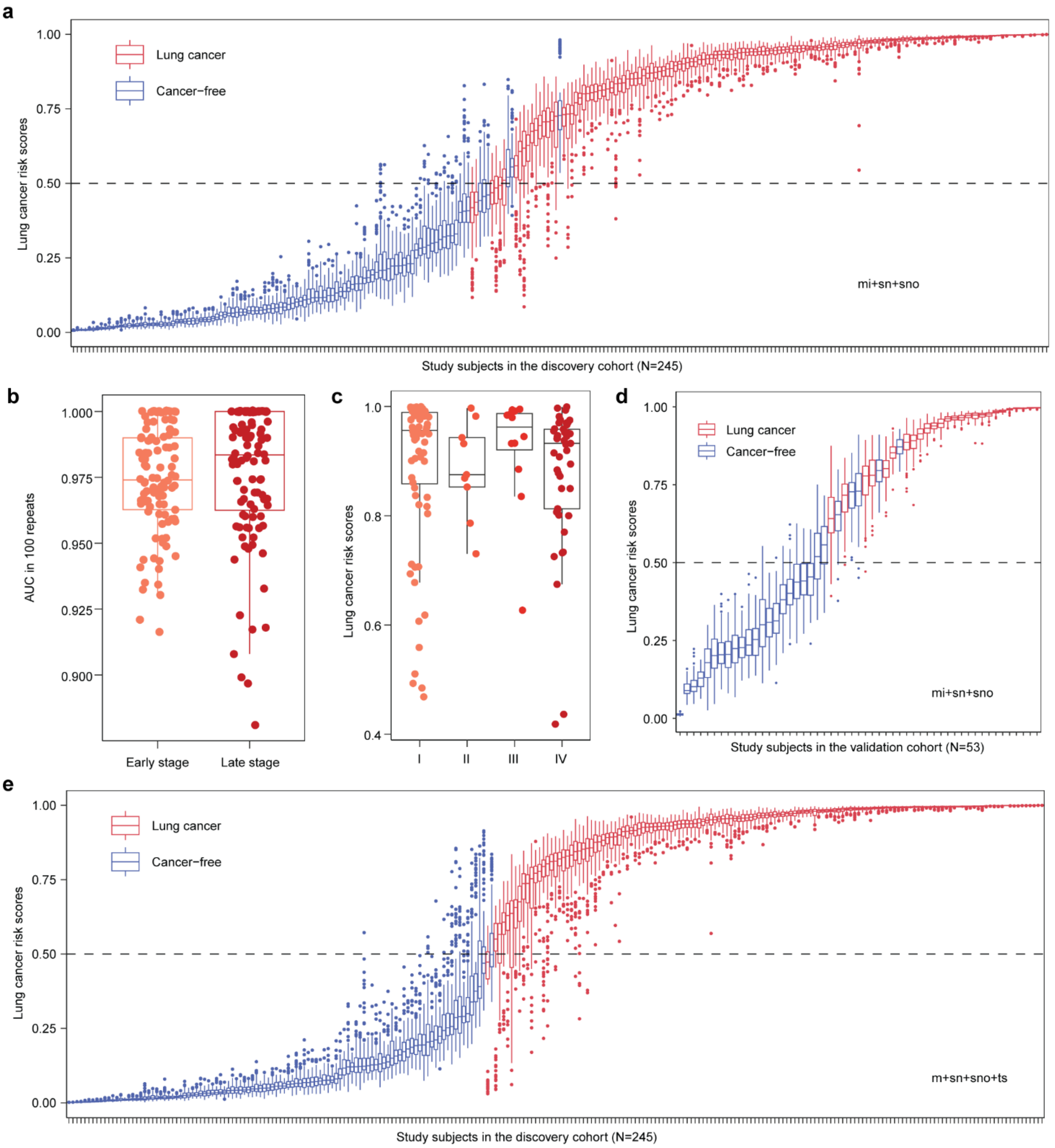

Supplementary Fig. 13 | a. Lung cancer risk scores of individual study subjects in the discovery cohort (N=245) predicted by 100 iterations of LR models trained with the “mi+sn+sno” panel. **b.** AUC over 100 repeats between early-stage and late-stage LC cancer patients predicted by LR models trained with the “mi+sn+sno” panel. **c.** Risk scores predicted by the “mi+sn+sno” panel in LC patients with different stages (according to the AJCC/UICC 7th Edition). **d.** Risk scores of individuals in the validation cohort (N=53) predicted by LR models trained with the “mi+sn+sno” panel. **e.** Risk scores of individuals in the discovery cohort predicted by LR models trained with the “m+sn+sno+ts” panel. The extremes of boxes define the upper and lower quartiles, and the center lines define the median. Whiskers indicate 1.5× interquartile range (IQR).

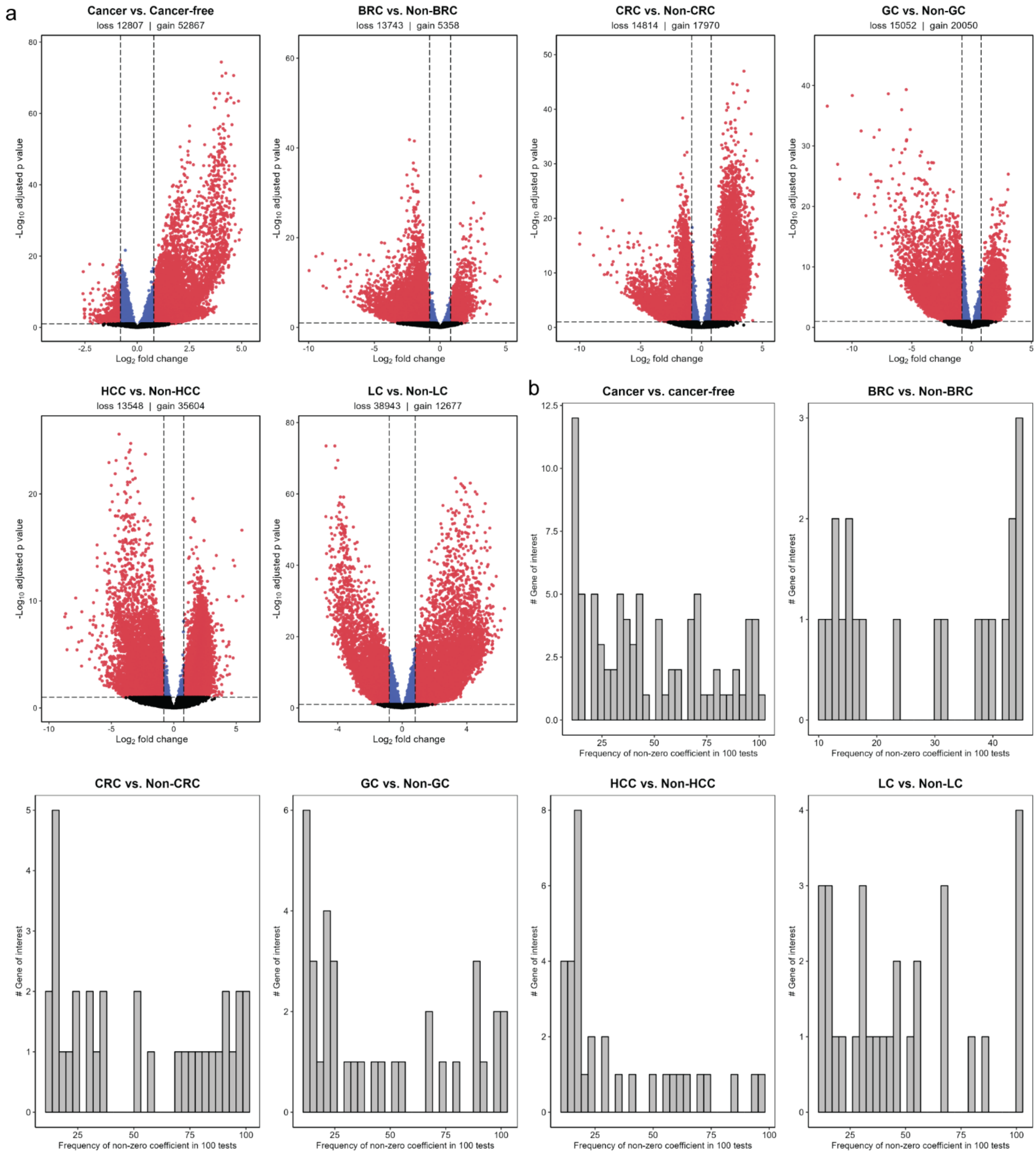

Supplementary Fig. 14 | a. Volcano plots showing six sets of one-class versus other-classes differential expression tests. Red dots indicate cfRNAs at BH-FDR < 0.1 (negative binomial GLM, Wald test, two-sided P values) and absolute $\log_2$ fold change > 0.8. **b.** Histograms showing the frequency of non-zero coefficient frequency in 100 model iterations in six sets of one-class versus other-classes LASSO regularized LR models training with cfRNAs of interest (mRNAs, miRNAs, snRNAs, snoRNAs, and tsRNAs).

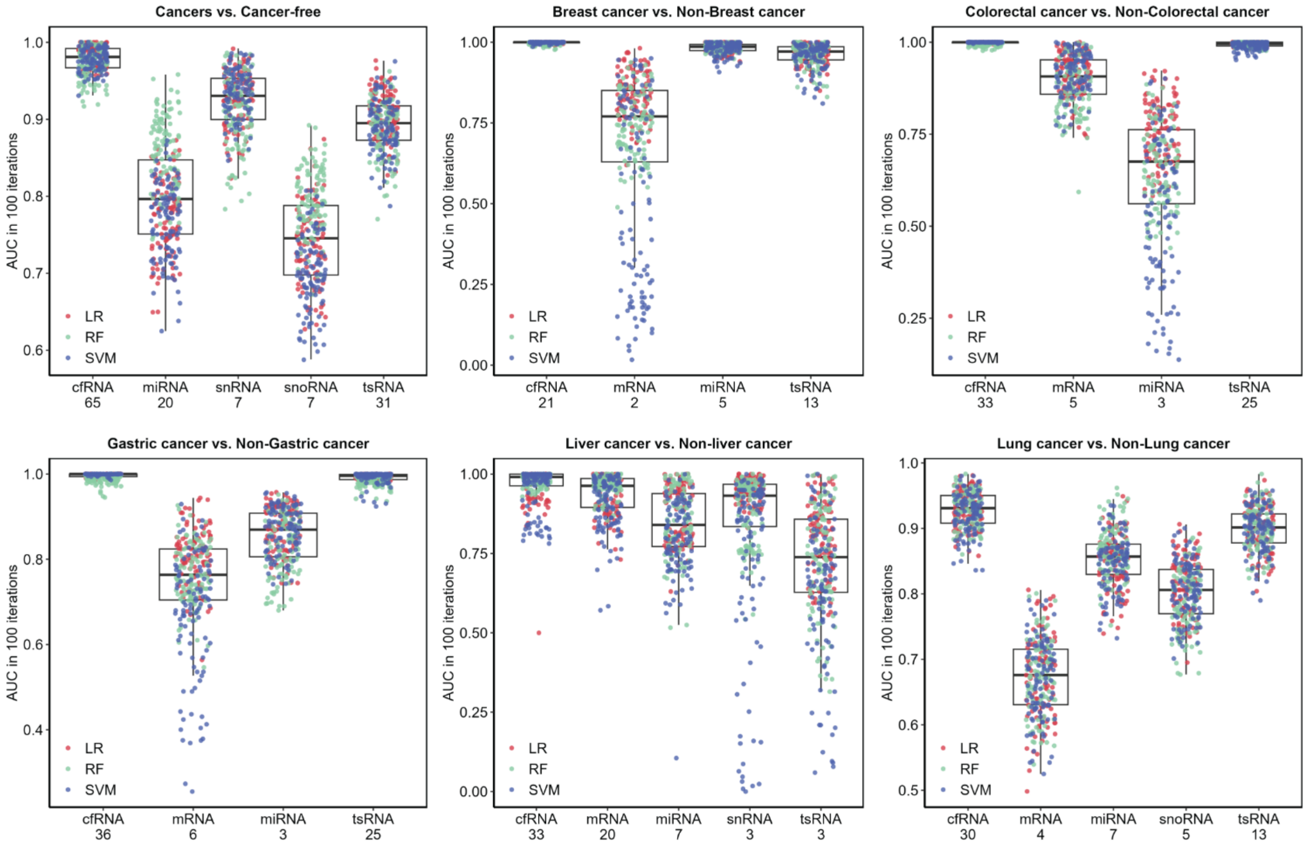

Supplementary Fig. 15 | AUC over 100 iterations of six sets of one-class versus other-classes machine learning analyses using three different machine learning classifiers (LR, RF, SVM). The AUC results for each RNA type are shown. The extremes of boxes define the upper and lower quartiles, and the center lines define the median. Whiskers indicate 1.5× interquartile range (IQR).

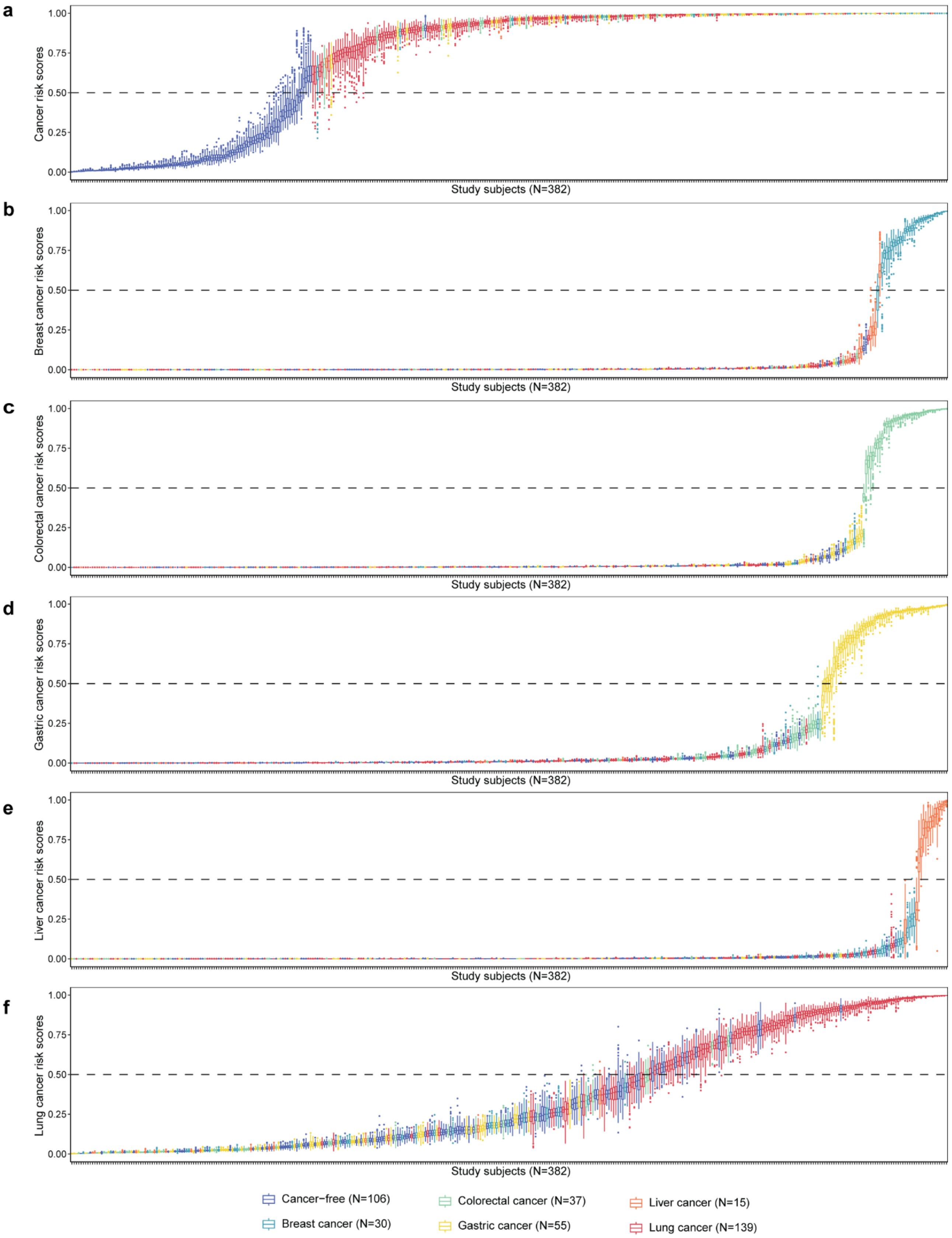

Supplementary Fig. 16 | Risk scores of individual study subjects in the discovery cohort (N=245) and pan-cancer cohort (N=137) predicted by 100 iterations of LR models trained with a 65-cfRNA cancer detection panel (**a**) and five cancer type-specific detection panels (21-cfRNA BRC detection panel (**b**), 33-cfRNA CRC detection panel (**c**), 36-cfRNA GC detection panel (**d**), 33-cfRNA HCC detection panel (**e**), 30-cfRNA LC detection panel (**f**)). The extremes of boxes define the upper and lower quartiles, and the center lines define the median. Whiskers indicate 1.5× interquartile range (IQR).

Supplementary Table

Supplementary Table 1 | Median AUC over 100 model iterations of three dissimilar machine learning classifiers (LR, RF, SVM) in the 80% training (a) and 20% test set (b) of the discovery cohort and the validation cohort (c). The results of different types of cfRNAs selected by three feature selection methods (Top N, Boruta, LASSO) are shown. The values with yellow background represent the number of candidate features used in the models.

a

AUC in the discovery cohort - training set										
Method	Classifier	mRNA	lncRNA	miRNA	piRNA	snRNA	snoRNA	tsRNA	rsRNA	ysRNA
Before selection		55	17	65	74	16	42	113	1114	104
Top N	Features	43	12	25	32	14	24	74	8	79
	LR	0.967	0.863	0.910	0.957	0.940	0.932	0.977	0.945	0.979
	RF	1.000	1.000	1.000	1.000	1.000	1.000	1.000	1.000	1.000
	SVM	0.983	0.870	0.917	0.963	0.952	0.948	0.995	0.950	0.996
Boruta	Features	42	14	46	55	16	29	68	152	59
	LR	0.951	0.878	0.919	0.969	0.951	0.921	0.969	1.000	0.956
	RF	1.000	1.000	1.000	1.000	1.000	1.000	1.000	1.000	1.000
	SVM	0.970	0.885	0.942	0.985	0.960	0.932	0.986	1.000	0.978
LASSO	Features	29	6	26	28	10	19	38	50	25
	LR	0.971	0.853	0.929	0.967	0.952	0.947	0.976	1.000	0.953
	RF	1.000	1.000	1.000	1.000	1.000	1.000	1.000	1.000	1.000
	SVM	0.981	0.854	0.939	0.969	0.960	0.951	0.983	1.000	0.954

b

AUC in the discovery cohort - test set										
Method	Classifier	mRNA	lncRNA	miRNA	piRNA	snRNA	snoRNA	tsRNA	rsRNA	ysRNA
Before selection		55	17	65	74	16	42	113	1114	104
Top N	Features	43	12	25	32	14	24	74	8	79
	LR	0.915	0.833	0.868	0.941	0.924	0.886	0.918	0.945	0.899
	RF	0.871	0.848	0.855	0.901	0.887	0.847	0.898	0.944	0.862
	SVM	0.920	0.828	0.866	0.936	0.928	0.893	0.887	0.945	0.900
Boruta	Features	42	14	46	55	16	29	68	152	59
	LR	0.899	0.843	0.836	0.938	0.933	0.857	0.908	0.990	0.882
	RF	0.856	0.832	0.838	0.915	0.907	0.834	0.893	0.968	0.862
	SVM	0.899	0.837	0.839	0.905	0.937	0.868	0.880	0.982	0.862
LASSO	Features	**29**	6	**26**	28	**10**	**19**	**38**	50	25
	LR	**0.935**	0.843	**0.885**	0.948	**0.942**	**0.910**	**0.933**	1.000	0.916
	RF	**0.885**	0.847	**0.851**	0.928	**0.904**	**0.867**	**0.919**	0.985	0.884
	SVM	**0.946**	0.834	**0.893**	0.935	**0.945**	**0.907**	**0.918**	1.000	0.910

c

AUC in the validation cohort										
Method	Classifier	mRNA	lncRNA	miRNA	piRNA	snRNA	snoRNA	tsRNA	rsRNA	ysRNA
Before selection		55	17	65	74	16	42	113	1114	104
Top N	Features	43	12	25	32	14	24	74	8	79
	LR	0.817	0.322	0.859	0.527	0.785	0.823	0.668	0.088	0.593
	RF	0.776	0.548	0.782	0.236	0.699	0.781	0.632	0.203	0.215
	SVM	0.672	0.299	0.837	0.634	0.829	0.826	0.651	0.162	0.548
Boruta	Features	42	14	46	55	16	29	68	152	59
	LR	0.755	0.252	0.876	0.553	0.853	0.789	0.660	0.679	0.447
	RF	0.750	0.551	0.730	0.229	0.769	0.744	0.641	0.519	0.200
	SVM	0.607	0.194	0.860	0.658	0.889	0.753	0.642	0.751	0.510
LASSO	Features	**29**	6	**26**	28	**10**	**19**	**38**	50	25
	LR	**0.846**	0.352	**0.905**	0.575	**0.843**	**0.788**	**0.719**	0.745	0.705
	RF	**0.821**	0.570	**0.759**	0.362	**0.759**	**0.733**	**0.701**	0.586	0.276
	SVM	**0.708**	0.315	**0.895**	0.578	**0.903**	**0.787**	**0.741**	0.819	0.793

Supplementary Table 2 | Pearson correlation coefficient between different cohorts and different RNA types.

	**mRNA**	**lncRNA**	**miRNA**	**piRNA**	**snRNA**	**snoRNA**	**tsRNA**	**rsRNA**	**ysRNA**	**cfRNA**
LC vs. NOR	0.996	0.997	0.995	0.992	0.996	0.983	0.994	0.966	0.990	0.987
BRC vs. GC	0.989	0.993	0.985	0.970	0.989	0.977	0.946	0.928	0.954	0.970
CRC vs. GC	0.985	0.987	0.995	0.969	0.995	0.984	0.986	0.938	0.978	0.966
BRC vs. HCC	0.982	0.981	0.995	0.989	0.989	0.968	0.986	0.925	0.985	0.956
BRC vs. NOR	0.991	0.991	0.983	0.970	0.979	0.960	0.969	0.921	0.957	0.953
GC vs. NOR	0.988	0.992	0.992	0.945	0.990	0.970	0.911	0.936	0.971	0.953
GC vs. LC	0.985	0.988	0.989	0.963	0.996	0.964	0.921	0.926	0.969	0.948
BRC vs. LC	0.986	0.986	0.988	0.981	0.987	0.965	0.971	0.917	0.974	0.947
BRC vs. CRC	0.968	0.980	0.983	0.974	0.982	0.953	0.914	0.868	0.910	0.924
CRC vs. LC	0.980	0.992	0.988	0.975	0.993	0.960	0.879	0.829	0.924	0.921
GC vs. HCC	0.969	0.976	0.979	0.974	0.985	0.973	0.930	0.841	0.939	0.920
CRC vs. NOR	0.977	0.991	0.990	0.961	0.989	0.982	0.862	0.818	0.932	0.914
HCC vs. NOR	0.970	0.965	0.982	0.962	0.983	0.978	0.956	0.884	0.958	0.880
HCC vs. LC	0.957	0.953	0.987	0.975	0.989	0.961	0.957	0.884	0.973	0.865
CRC vs. HCC	0.935	0.946	0.976	0.969	0.986	0.974	0.896	0.736	0.888	0.836

Supplementary Table 3 | Participant demographics and baseline characteristics of study cohorts.

	Discovery cohort		Validation cohort		Pan-cancer patient cohort			
	**LC**	**NOR**	**LC**	**NOR**	**BRC**	**CRC**	**GC**	**HCC**
**Total, n**	139	106	26	27	30	37	55	15
**Age, Mean ± SD**	56 ± 12	49 ± 12	44 ± 11	33 ± 9	54 ± 11	62 ± 12	59 ± 12	62 ± 10
**Sex, n (%)**								
**Male**	60 (43%)	51 (48%)	9 (35%)	16 (59%)	-	27 (73%)	33 (60%)	10 (67%)
**Female**	79 (57%)	55 (52%)	17 (65%)	11 (41%)	30 (100%)	10 (27%)	18 (33%)	5 (33%)
**Race, n (%)**								
**Asian**	139 (100%)	106 (100%)	26 (100%)	27 (100%)	30 (100%)	37 (100%)	55 (100%)	15 (100%)
**TNM Stage, n (%)**								
**I**	72 (52%)	-	26 (100%)	-	1 (3%)	7 (19%)	6 (11%)	-
**II**	9 (6%)	-	-	-	15 (50%)	15 (41%)	14 (25%)	4 (27%)
**III**	12 (9%)	-	-	-	7 (23%)	13 (35%)	24 (44%)	8 (53%)
**IV**	45 (32%)	-	-	-	5 (17%)	2 (5%)	1 (2%)	3 (20%)
**Missing**	1 (1%)	106 (100%)	-	27 (100%)	2 (7%)	0 (0%)	10 (18%)	0 (0%)
**Stage Group, n (%)**								
**Early**	81 (58%)	-	26 (100%)	-	16 (53%)	22 (59%)	20 (36%)	1 (7%)
**Late**	57 (41%)	-	-	-	12 (40%)	15 (41%)	25 (45%)	10 (67%)
**Missing**	1 (1%)	106 (100%)	-	27 (100%)	2 (7%)	0 (0%)	10 (18%)	0 (0%)

Supplementary Data

Supplementary Data 1. Primer design and average Cq values in the qPCR experiments.
Supplementary Data 2. Synthetic RNA and tissue specific genes detected by SLiPiR-seq and NEBNext.
Supplementary Data 3. ID and sequence of the established reference genome for rsRNAs and ysRNAs.
Supplementary Data 4. Results of differential expression analyses performed in this study.
Supplementary Data 5. Names of selected candidate RNAs in the cancer detection models.
Supplementary Data 6. Summary of different cfRNA combinations.
Supplementary Data 7. Risk scores of all studied individuals predicted by six cancer detection models.
Supplementary Data 8. Raw read count matrix for technology optimizations.
Supplementary Data 9. Raw read count matrix for all de-identified clinical samples.
