# Supplementary material for: Terminal modifications independent cell-free RNA sequencing enables sensitive early cancer detection and classification
Source: Nat Commun. 2024 Jan 2;15:156. doi: 10.1038/s41467-023-44461-y (PMC10761679; doi:10.1038/s41467-023-44461-y)
Supplement: Supplementary file 3 — Description of Additional Supplementary Files [file 41467_2023_44461_MOESM3_ESM.pdf]

Description of Additional Supplementary Files

Supplementary Data 1
Description: Primer design and average Cq values in the qPCR experiments.

Supplementary Data 2
Description: Synthetic RNA and tissue specific genes detected by SLiPiR-seq and NEBNext.

Supplementary Data 3
Description: ID and sequence of the established reference genome for rsRNAs and ysRNAs.

Supplementary Data 4
Description: Results of differential expression analyses performed in this study.

Supplementary Data 5
Description: Names of selected candidate RNAs in the cancer detection models.

Supplementary Data 6
Description: Summary of different cfRNA combinations.

Supplementary Data 7
Description: Risk scores of all studied individuals predicted by six cancer detection models.

Supplementary Data 8
Description: Raw read count matrix for technology optimizations.

Supplementary Data 9
Description: Raw read count matrix for all de-identified clinical samples.
