# Supplementary material for: Terminal modifications independent cell-free RNA sequencing enables sensitive early cancer detection and classification
Source: Nat Commun. 2024 Jan 2;15:156. doi: 10.1038/s41467-023-44461-y (PMC10761679; doi:10.1038/s41467-023-44461-y)
Supplement: Supplementary file 13 — Reporting Summary [file 41467_2023_44461_MOESM13_ESM.pdf]

nature portfolio

Corresponding author(s): Deming Gou

Last updated by author(s): Nov 16, 2023

Reporting Summary

Nature Portfolio wishes to improve the reproducibility of the work that we publish. This form provides structure for consistency and transparency in reporting. For further information on Nature Portfolio policies, see our Editorial Policies and the Editorial Policy Checklist.

Statistics

For all statistical analyses, confirm that the following items are present in the figure legend, table legend, main text, or Methods section.

n/a	Confirmed	
☐	☒	The exact sample size (*n*) for each experimental group/condition, given as a discrete number and unit of measurement
☐	☒	A statement on whether measurements were taken from distinct samples or whether the same sample was measured repeatedly
☐	☒	The statistical test(s) used AND whether they are one- or two-sided<br>*Only common tests should be described solely by name; describe more complex techniques in the Methods section.*
☐	☒	A description of all covariates tested
☐	☒	A description of any assumptions or corrections, such as tests of normality and adjustment for multiple comparisons
☐	☒	A full description of the statistical parameters including central tendency (e.g. means) or other basic estimates (e.g. regression coefficient) AND variation (e.g. standard deviation) or associated estimates of uncertainty (e.g. confidence intervals)
☐	☒	For null hypothesis testing, the test statistic (e.g. *F*, *t*, *r*) with confidence intervals, effect sizes, degrees of freedom and *P* value noted<br>*Give P values as exact values whenever suitable.*
☒	☐	For Bayesian analysis, information on the choice of priors and Markov chain Monte Carlo settings
☐	☒	For hierarchical and complex designs, identification of the appropriate level for tests and full reporting of outcomes
☐	☒	Estimates of effect sizes (e.g. Cohen's *d*, Pearson's *r*), indicating how they were calculated

Our web collection on statistics for biologists contains articles on many of the points above.

Software and code

Policy information about availability of computer code

Data collection	No software was used for data collection.
Data analysis	All computational analyses were performed using Linux shell, Python 3, or R 4.2. Scripts used to generate the findings in this study are available on Github at https://github.com/JinyongHuang/SLiPiR-seq.<br>Software version:<br>cutadapt (version 2.10); trimmomatic (version 0.39); bowtie2 (version 2.4.4); subread package (version 2.0.3);<br>python (version 3.9.0);<br>R (version 4.2.1); tidyverse (version 2.0.0); ggpubr (version 0.6.0); ggplot2 (version 3.4.2); ggpmisc (version 0.5.2); ggrepel (version 0.9.3);<br>RColorBrewer (version 1.1-3); patchwork (version 1.1.2); DESeq2 (version 1.34.0); EnhancedVolcano (version 1.12.0); caret (version 6.0-94);<br>foreach (version 1.5.2); doParallel (version 1.0.17); glmnet (version 4.1-7); randomForest (version 4.7-1.1); ROCR (version 1.0-11);<br>pROC (version 1.18.0); reshape2 (version 1.4.4)

For manuscripts utilizing custom algorithms or software that are central to the research but not yet described in published literature, software must be made available to editors and reviewers. We strongly encourage code deposition in a community repository (e.g. GitHub). See the Nature Portfolio guidelines for submitting code & software for further information.

Data

Policy information about availability of data

All manuscripts must include a data availability statement. This statement should provide the following information, where applicable:

- Accession codes, unique identifiers, or web links for publicly available datasets
- A description of any restrictions on data availability
- For clinical datasets or third party data, please ensure that the statement adheres to our policy

Raw sequencing data (fastq) and raw read count matrix for technology optimizations are available through the Sequence Read Archive (SRA, PRJNA962827, https://www.ncbi.nlm.nih.gov/sra) and Supplementary Data 8, respectively. Raw read count matrices for all detected cfRNAs and all de-identified studied clinical samples are available at Supplementary Data 9. The raw sequencing data for clinical samples reported in this paper have been deposited in the Genome Sequence Archive in Natonal Genomics Data Center, China National Center for Bioinformation / Beiing Institute of Genomics, Chinese Academy of Sciences (GSA-Human: HRA004959, https://ngdc.cncb.ac.cn/gsa-human). The raw sequencing data are under controlled access and are available upon request from the corresponding author to comply with ethics regulation of Shenzhen University. Applicants should have obtained ethical approvals from their ethics committees and submitted a reasonable research proposal for the data request. Timescale for access to be granted would be around one month and there are no restrictions on duration of access.

Reference genome: miRNA (miRbase, https://www.mirbase.org/), piRNA (piRNABank, http://pirnabank.ibab.ac.in/), tsRNA (MINTbase, https://cm.jefferson.edu/MINTbase/), GRCh38 (hg38, https://hgdownload.soe.ucsc.edu/goldenPath/hg38/bigZips/), GENCODE (https://www.gencodegenes.org/human/stats_41.html)

Research involving human participants, their data, or biological material

Policy information about studies with human participants or human data. See also policy information about sex, gender (identity/presentation), and sexual orientation and race, ethnicity and racism.

Reporting on sex and gender	Sex and gender are not considered as covariate in the study design. Sample recruitment has no bias on sex and gender. The sex and gender distributions of our study cohorts are available in Extended Data Table 2.
Reporting on race, ethnicity, or other socially relevant groupings	All participants included in this study are Asian. This is because all participants were recruited from different clinical centers in China, where the population demographics are predominantly Asian.
Population characteristics	The cohorts described herein are composed of cancer-free donors and cancer patients of varied age, cancer types and TNM stage characteristics (Extended Data Table 2).
Recruitment	This is a retrospective study of samples collected from five different clinical centers. Study subjects were recruited at People's Hospital of Bao'an Shenzhen, The Fifth People's Hospital of Suzhou, The Second People's Hospital of Shenzhen, Peking University Shenzhen Hospital, General Hospital of Ningxia Medical University. The study protocol was reviewed and approved by the Institutional Review Board of participating sites. Informed written consent was obtained for each participant prior to sample collection. Liquid biopsy samples were collected either at the time of surgery (prior to tumor removal) or during routine follow-up at the participating sites. We took care to avoid any potential bias or self-selection issues that could have that may have affected our retrospective study design.
Ethics oversight	Our research complies with ethical regulations within all participating sites. This project was approved by the Institutional Review Board at People's Hospital of Bao'an Shenzhen, the Institutional Review Board at The Fifth People's Hospital of Suzhou, the Institutional Review Board at The Second People's Hospital of Shenzhen, the Institutional Review Board at Peking University Shenzhen Hospital, the Institutional Review Board at General Hospital of Ningxia Medical University.

Note that full information on the approval of the study protocol must also be provided in the manuscript.

Field-specific reporting

Please select the one below that is the best fit for your research. If you are not sure, read the appropriate sections before making your selection.

[x] Life sciences [ ] Behavioural & social sciences [ ] Ecological, evolutionary & environmental sciences

For a reference copy of the document with all sections, see nature.com/documents/nr-reporting-summary-flat.pdf

Life sciences study design

All studies must disclose on these points even when the disclosure is negative.

Sample size	Study subjects were recruited at People's Hospital of Bao'an Shenzhen (N=111 cancer-free individuals, NOR_SZBA), The Fifth People's Hospital of Suzhou (N=27 cancer-free individuals, NOR_SZDW), The Second People's Hospital of Shenzhen (N=140 lung cancer patients, LC_SZDE), Peking University Shenzhen Hospital (N=28 lung cancer patients, LC_SZBU), General Hospital of Ningxia Medical University (N=30 breast cancer patients, BRC_NXYK; N=37 colorectal cancer patients, CRC_NXYK; N=55 gastric cancer patients, GC_NXYK; and N=16 liver cancer patients, HCC_NXYK). No statistical method was used to predetermine the sample size. The sample size for this study was determined based on the number of participants that could be recruited from each clinical center. Based on the results from a small scale pilot study, the sample size used in this study is sufficient for the identification of differentially expressed cfRNAs and the training and validation of machine learning models.
Data exclusions	Exclusion criteria of low-quality samples are: (1) Clean reads ratio < 20%; (2) Clean reads < 2 million; (3) rsRNA ratio > 30%; (4) the sum of

	lsRNA and msRNA ratio > 30%. 9 of 444 samples were excluded based on these criteria.
Replication	All experiments for the technology optimizations of SLiPiR-seq were repeated at least three times to ensure reproducibility. All sample partitions and model training and testing processes were repeated 100 times to avoid random sampling biases. All attempts at replication were successful and the results can be reproduced.
Randomization	The discovery cohort was randomly partitioned into training and test sets in an 80–20% manner using the R package caret. The training set was used to train models and the held-out test set was used to assess the models. The sample partitioning and training-test processes were repeated 100 times for all models to avoid bias.
Blinding	Experiments for the technology optimizations were not blinded as all tested conditions should be cleary labeled to avoid confusion during experiments. For the case-control study, samples were blinded during cfRNA extraction, library preparation and sequencing. Samples were unblinded during data processing and analysis.

Reporting for specific materials, systems and methods

We require information from authors about some types of materials, experimental systems and methods used in many studies. Here, indicate whether each material, system or method listed is relevant to your study. If you are not sure if a list item applies to your research, read the appropriate section before selecting a response.

Materials & experimental systems

n/a	Involved in the study
☒	☐ Antibodies
☒	☐ Eukaryotic cell lines
☒	☐ Palaeontology and archaeology
☒	☐ Animals and other organisms
☒	☐ Clinical data
☒	☐ Dual use research of concern
☒	☐ Plants

Methods

n/a	Involved in the study
☒	☐ ChIP-seq
☒	☐ Flow cytometry
☒	☐ MRI-based neuroimaging
