# Supplementary material for: Terminal modifications independent cell-free RNA sequencing enables sensitive early cancer detection and classification
Source: Nat Commun. 2024 Jan 2;15:156. doi: 10.1038/s41467-023-44461-y (PMC10761679; doi:10.1038/s41467-023-44461-y)
Supplement: Supplementary file 2 — Peer Review File [file 41467_2023_44461_MOESM2_ESM.pdf]

nature portfolio

Peer Review File

Terminal modifications independent cell-free RNA sequencing enables sensitive early cancer detection and classification

Open Access This file is licensed under a Creative Commons Attribution 4.0 International License, which permits use, sharing, adaptation, distribution and reproduction in any medium or format, as long as you give appropriate credit to the original author(s) and the source, provide a link to the Creative Commons license, and indicate if changes were made. In the cases where the authors are anonymous, such as is the case for the reports of anonymous peer reviewers, author attribution should be to 'Anonymous Referee' followed by a clear attribution to the source work. The images or other third party material in this file are included in the article's Creative Commons license, unless indicated otherwise in a credit line to the material. If material is not included in the article's Creative Commons license and your intended use is not permitted by statutory regulation or exceeds the permitted use, you will need to obtain permission directly from the copyright holder. To view a copy of this license, visit http://creativecommons.org/licenses/by/4.0/.

REVIEWER COMMENTS

Reviewer #1 (Remarks to the Author): expertise in RNA subtypes

The authors tried to develop a sensitive library preparation method that could analyze the profiling of cfRNAs independent of their 5′-end modifications in plasma. They have conducted transcriptome-wide characterizations in patients with various cancer types and cancer-free donors, coupled with a bioinformatics analysis pipeline and machine learning analyses, demonstrating its ability to detect transcriptomic changes occurring in early-stage tumors.

Some of my concerns are listed as follows:
1, In the methods of reads calling for tsRNAs, rsRNAs and ysRNAs, the full-length Homo sapiens ribosomal RNAs and Y RNAs sequences from NCBI were used for alignment, however, reference genome of tsRNA were obtained from the MINTbase. why not use the Homo sapiens transfer RNAs sequences, or the reference genome of tRNA that from wellknown GtRNAdb database (http://gtrnadb.ucsc.edu/).

2, Line 380-383, in the characterizations of tsRNAs, rsRNAs and ysRNAs, fragments with identical sequences for the first 15 nucleotides from the 5' end were treated as one RNA species. The sequences of the subspecies with the longest fragment size were archived into the reference genome under construction.
It is interesting, read counts of all subspecies was summed to represent the counts of one RNA species. But, sometimes, the length of subspecies fragments varies between 16bp and 50bp in one RNA species (Supplementary Data 3), this issue is worth thinking about deeply.

3, In the Fig.3d-f, the visualization of read coverage profiles of diverse cfRNA, the vertical axis indicate the RPM values. However, there are large variation in the range of scale values, with different index number of x10, it is a little confusing for me. What does it mean differently? Could you use a unified index number?

4, In the legend, there is no clear definition and description for some abbreviations, such as “R1” and “R2” in Fig.S1, “5′-tRHs”, “i-tRFs” and “3′-tRFs” in Fig.3c, “AUC” in Fig.5a. The aim of the legend should be to describe the key messages of the figure, but the figure should also be discussed in the text. The legend itself should be succinct, while still explaining all symbols and abbreviations. May be, you should try to describe the mean of abbreviations in the legend of figure or in the supplementary materials.

5, There are many descriptions for the categories of tsRNA, such as “5′-tRHs”, “i-tRFs”, “3′-tRFs” and “3′-tRHs” in the manuscript, and other abbreviations in previous papers. Meanwhile, the author should discuss the advantage and scientific merit of tsRNAs over other previously explored biomarkers. Some relevant citation could be found, "DOI:10.1155/2022/8708312 ".

6, Minor comments: The authors should revise English writing carefully and eliminate some errors in the paper to make the paper easier to read.

Reviewer #2 (Remarks to the Author): expertise in cfRNA detection methods (wet lab)

The research manuscript titled "Terminal modifications independent cell-free RNA sequencing enables sensitive early cancer detection and classification" by Jun Wang and colleagues presents a novel and insightful approach for future investigations of small RNAs in cancer. The manuscript explores the potential of small RNAs as diagnostic biomarkers in various cancer. MiRNAs as potential diagnostic biomarkers have been intensively explored in various diseases, including cancer. Most of the commercially available small RNA library preparation kits are adaptor ligation-based which need the presence of a 3' hydroxyl and 5' phosphate group. As miRNAs are typically 5′ phosphorylated and 3′ hydroxylated, therefore, these kits were mostly used for miRNA sequencing. To address this obstacle, the authors developed the innovative Splint Ligation and Phosphate-Independent RNA Sequencing (SLiPiR-seq) method, which allows for the construction of

libraries using small RNAs regardless of their 5′ phosphorylation status. The applicability of SLiPiR-seq was further validated using synthetic small RNAs, both with and without a 5′-P group, and was compared with a commercially available adapter ligation-based kit (NEBNext Small RNA Library Prep Set) in detecting cfRNAs in human plasma samples. The ability of SLiPiR-seq in detecting a broader coverage of the cfRNA repertoire will enable investigation of the functional roles and diagnostic potential of these small RNAs in various disease. Furthermore, the authors should be commented for their excellent work in establishing a robust bioinformatics analysis pipeline and implementing machine learning models to detect and localize different types of cancers using the small RNA sequencing data. In conclusion, I recommend the publication of this manuscript in the journal.

As SLiPiR-seq literally could sequence any fragmented RNA, it will bring challenge in following analysis of the sequenced data. However, these challenges could also be opportunity in developing new analysis protocols and identifying new diagnostic markers using these under investigated cfRNAs. Additionally, some minor issues in grammar need to be addressed before final acceptance as shown below.

Reviewer #3 (Remarks to the Author): expertise in cfRNA bioinformatics

1. What are the noteworthy results?

The authors described a novel method for analyzing cell-free RNAs (cfRNAs) in plasma. The major novelty of the article is:

• The optimisation of SLiPiR-seq, a library preparation method that is sensitive to cfRNAs with different 5′-end modifications and requires only 100 μl of input plasma.
• The transcriptome-wide characterization of cfRNAs in 165 lung cancer, 30 breast cancer, 37 colorectal cancer, 55 gastric cancer, 15 liver cancer, and 133 cancer-free participants, revealing transcriptomic changes in early-stage tumors.
• The application of machine learning analyses on the differentially expressed cfRNA signatures, demonstrating their robust performance in cancer detection and classification.

2. Will the work be of significance to the field and related fields? How does it compare to the established literature? If the work is not original, please provide relevant references.

To the best of my knowledge, the author is the first group that demonstrated SLiPiR-seq can capture more RNA species than conventional adapter ligation-based methods. The authors also provided extensive technical characterisation of SLiPiR-seq data through clear visualisations of sequencing metrics and comparisons with canonical methods.

However, the SLiPiR-seq methodology described in this study is not significantly different from the method previously described by Maguire et al. Both methods use splint ligation as a key step to ligate adapters to RNA fragments without requiring a 5′-phosphate group. Maguire's team compared SLiPiR-seq against Illumina's TruSeq, New England Biolab's NEBNext, Perkin Elmer's NEXTflex methods, while the current study only compared SLiPiR-seq against NEBNext.
Maguire, S., Lohman, G. J., & Guan, S. (2020). A low-bias and sensitive small RNA library preparation method using randomized splint ligation. Nucleic acids research, 48(14), e80-e80.

The method developed by Maguire et al was based on the following works.
Nilsen T. W. (2013). Splinted ligation method to detect small RNAs. Cold Spring Harbor protocols, 2013(1), pdb.prot072611. https://doi.org/10.1101/pdb.prot072611
Jin, J., Vaud, S., Zhelkovsky, A. M., Posfai, J., & McReynolds, L. A. (2016). Sensitive and specific miRNA detection method using SplintR Ligase. Nucleic acids research, 44(13), e116. https://doi.org/10.1093/nar/gkw399

Proper discussion and citation of the above works would allow readers differentiate this work from established literature. Besides, the protocol optimisations made by the authors, presumably

allowing the method to be more sensitive to low quantity of cfRNA, should be clearly highlighted in the main text.

3. Does the work support the conclusions and claims, or is additional evidence needed?

In line 115, the authors noted that "the fragment size of msRNAs and lsRNAs exhibited smoother distribution and broader coverage (23-60 nt)". Could the authors apply appropriate statistical tests to substantiate the claim?

In line 170, the authors noted "a significant increase in the cumulative expression level in early stage LC patients compared to controls". Could this observation due to confounding factors, bias in normalisation or multi-mapped reads? A recent study found that common normalization methods vary across different datasets.
Düren, Y., Lederer, J., & Qin, L. X. (2022). Depth normalization of small RNA sequencing: using data and biology to select a suitable method. Nucleic acids research, 50(10), e56.
https://doi.org/10.1093/nar/gkac064

In line 185-261, the authors attempted to use SLiPiR-seq data to develop a cfRNA signature for early detection of cancer. However, given by the relatively small sample size and diverse cancer types, it is unclear if the study has enough statistical power to substantiate the claim in line 260. "Collectively, these results demonstrate that cfRNA signatures identified by SLiPiR-seq can precisely discriminate different cancer types".

4. Are there any flaws in the data analysis, interpretation and conclusions? Do these prohibit publication or require revision?

In Fig. 2a, it looks like the dots corresponding to miRNAs are all below the trend line. Could it mean that miRNA expression is most likely underestimated by SLiPiR-seq?

In line 140, the authors classified tsRNAs, rsRNAs and ysRNAs species based on the first 15 nucleotides. I would expect more comprehensive comparison of RNA species using established databases such as Rfam and RNAcentral.

In Fig. 4e, the legends are unclear. The colour blocks below the dendrogram, on the left of the heatmap, at the bottom of the heatmap should be clearly labelled. Description of blocks in grey is not defined.

In line 168, should absolute log2 fold change < 1 be used instead?

The surprisingly high sensitivity of 96.15% and specificity of 100% may indicate overfitting under high dimensionality of the features. While it may not be feasible to have independent training, testing and validation samples, the use of regularisation techniques and cross-validation may alleviate overfitting of features.

5. Is the methodology sound? Does the work meet the expected standards in your field?
Yes in general, but please note the flaws above.

6. Is there enough detail provided in the methods for the work to be reproduced?
Yes

RESPONSE TO REVIEWERS' COMMENTS

Reviewer #1

Question1: In the methods of reads calling for tsRNAs, rsRNAs and ysRNAs, the full-length Homo sapiens ribosomal RNAs and Y RNAs sequences from NCBI were used for alignment, however, reference genome of tsRNA were obtained from the MINTbase. why not use the Homo sapiens transfer RNAs sequences, or the reference genome of tRNA that from wellknown GtRNAdb database (http://gtrnadb.ucsc.edu/).

Answer: We appreciate the reviewer raising the question about our rationale for using MINTbase rather than GtRNAdb or Homo sapiens transfer RNA sequences for characterization of tsRNA in plasma. To provide robust quantification and classification of tsRNA profiles, we opted to use MINTbase over GtRNAdb for the following reasons:

1) When aligning to rRNA and Y RNA reference genomes, approximately 95% of mapped reads were unique alignments. However, due to the highly similar sequences for the ~600 tRNAs, mapping to GtRNAdb resulted in only ~1% uniquely aligned reads, with ~14% multiple mapped reads (Table 1). The high rate of multi-mapping with GtRNAdb precludes reliable quantification and classification of individual tRNA species.

Table 1 Reads mapped to the GtRNAdb tRNA reference genome (hg38-tRNAs.fa).

	Total clean reads	Unique mapped reads	Multiple mapped reads
SLiPiR-seq	20M	~ 0.23M (1.13%)	~ 2.84M (14.19%)
NEBNext	20M	~ 0.002M (0.01%)	~ 0.08M (0.40%)

2) MINTbase contains over 28,824 non-redundant, validated tsRNA sequences with comprehensive annotations (length, category, amino acid, anticodon, source, etc). This allowed robust classification of plasma tsRNA profiles not possible by alignment to full-length tRNAs in GtRNAdb.

Pliatsika, V. *et al.* MINTbase v2.0: a comprehensive database for tRNA-derived fragments that includes nuclear and mitochondrial fragments from all The Cancer Genome Atlas projects. *Nucleic Acids Res* **46**, D152-D159, doi:10.1093/nar/gkx1075 (2018).

3) Critically, when using MINTbase for tsRNA quantification, we were able to classify nearly all (14% vs 15%) of the reads aligning to the tRNA genome (hg38-tRNAs.fa). This indicates that MINTbase captures the majority of authentic tsRNA sequences in plasma.

In summary, due to the sequence similarity among tRNAs, alignment to a database of validated tsRNA sequences in MINTbase enables more accurate quantification and classification compared to alignment to full-length tRNAs. The exhaustively curated MINTbase tsRNA reference provides a more suitable foundation for characterization of the plasma tsRNA repertoire. We agree with the reviewer that justification of our database choice will add rigor to our tsRNA quantification bioinformatic analysis, and we added "Due to the high similarity of the sequences of parent tRNAs, alignment to a database of validated tsRNA sequences allows for more accurate quantification than alignment to a database of full-length tRNAs." to the method section to validate our choice.

Question2: Line 380-383, in the characterizations of tsRNAs, rsRNAs and ysRNAs, fragments with identical sequences for the first 15 nucleotides from the 5' end were treated as one RNA species. The sequences of the subspecies with the longest fragment size were archived into the reference genome under construction.

It is interesting, read counts of all subspecies was summed to represent the counts of one RNA species. But, sometimes, the length of subspecies fragments varies between 16bp and 50bp in one RNA species (Supplementary Data 3), this issue is worth thinking about deeply.

Answer: We agree with the reviewer's concern about the classification of RNA species. We have removed the analytical step that classifies fragments with identical sequences for the first 15 nucleotides from the 5' end as one RNA species. To avoid too many RNA species, we also tweaked the codes for bowite2 alignment to tolerate zero mismatch. As a result, we detected a total of 45397 rsRNA and 2664 ysRNA unique sequences.

However, the revision on the classification of RNA species inevitably altered other results demonstrated in the paper, resulting in extensive modifications. For example, the number of detected RNA species for tyr-sRNA in fig. 1c and fig. 2d were updated. Fig. 3a was also updated.

After revision, a total of 152858 cfRNA species were used for the differential expression analysis, which is 1.5 times more than before. This will affect the normalization of the cfRNA read count matrix and the calculation of adjusted p values during the DESeq2 analysis. Therefore, all results from fig. 4 to fig. 6 and their supplementary figures were inevitably changed. After revision, rsRNA accounts for 73.4% of all differentially expressed cfRNA and overshadows the performance of other RNA types. To compensate for this issue, we adjusted the inclusion criteria of candidate cfRNA selection from log2 fold change>1 to >0.8, which allows more cfRNAs of other types to be applied for the subsequent feature selection test.

Overall, although most results were updated, the inclusion of more tyr-sRNA species does not alter the findings and conclusions of our study. Interestingly, LR and SVM models training with 50 rsRNA (SVM AUC= 0.819 [0.786-0.843]) or 25 ysRNA (SVM AUC= 0.793 [0.747-0.829]) features showed high AUC in LC detection. However, models trained by the RF algorithm shared no agreement with these results (Supplementary Fig. 11c). Therefore, rsRNA and ysRNA were not used in the combination test. Again, the five RNA types of interest (mRNAs, miRNAs, snRNAs, snoRNAs, and tsRNAs) were used in the combination test and the cancer classification test.

Question3: In the Fig.3d-f, the visualization of read coverage profiles of diverse cfRNA, the vertical axis indicate the RPM values. However, there are large variation in the range of scale values, with different index number of x10, it is a little confusing for me. What does it mean differently? Could you use a unified index number?

Answer: We appreciate the reviewer raising this point about the inconsistent scaling between panels in Figures 3d-f, which was unintentionally confusing. To provide a clear comparison of the read coverage profiles between SLiPiR-seq and NEBNext for the different RNA types, we have revised the figures (shown below) to display the data for both methods in each panel with a unified y-axis scale. The updated figures now clearly show the difference in read coverage depth and breadth between the two methods on the same scale. For example, the much greater sequencing depth achieved by SLiPiR-seq compared to NEBNext for 5S rRNA, 5.8S rRNA,

18S rRNA, 28S rRNA, genomic tRNA, mitochondrial tRNA and RNY5 is now evident in the revised figure with the unified scale.

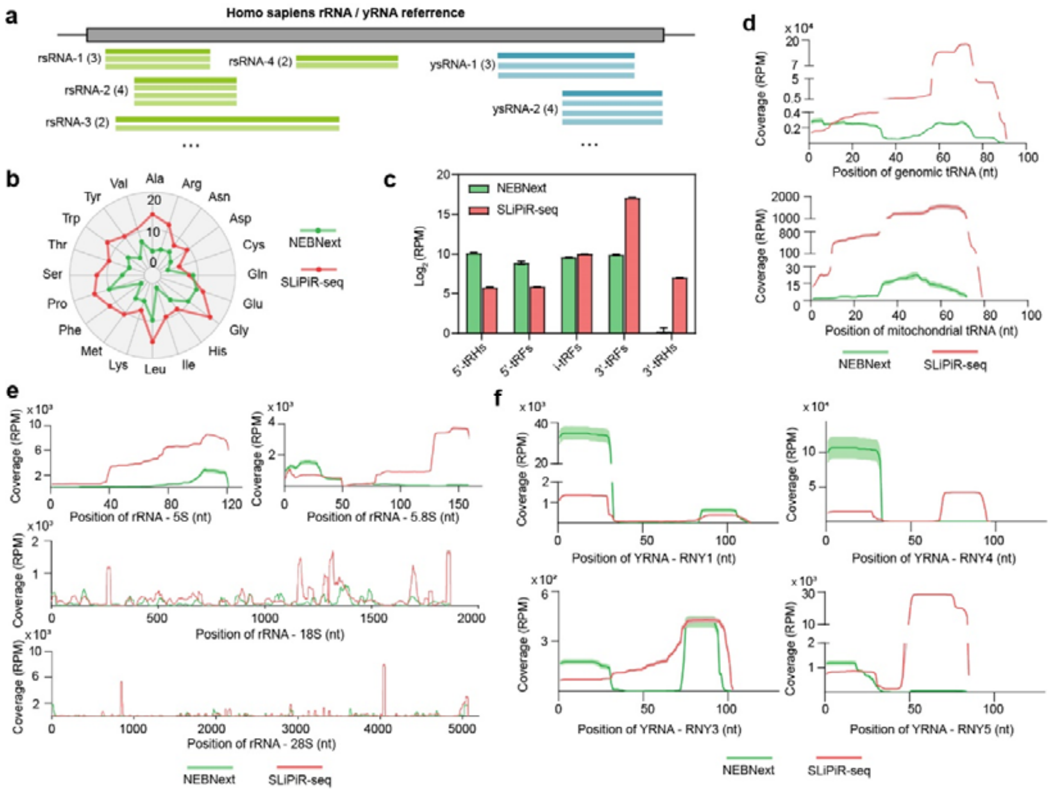

Question4: In the legend, there is no clear definition and description for some abbreviations, such as “R1” and “R2” in Fig.S1, “5’-tRHs”, “i-tRFs” and “3’-tRFs” in Fig.3c, “AUC” in Fig.5a. The aim of the legend should be to describe the key messages of the figure, but the figure should also be discussed in the text. The legend itself should be succinct, while still explaining all symbols and abbreviations. May be, you should try to describe the mean of abbreviations in the legend of figure or in the supplementary materials.

Answer: We appreciate the reviewer raising this important point about clearly defining abbreviations and symbols used in our figures and legends. As suggested, we have thoroughly reviewed all figure legends to ensure abbreviations and acronyms are defined at first use.

For example, in the caption of Figure S1, we added “R1 and R2 indicate the forward reads and reverse reads from paired-end sequencing, respectively.”. In the caption of Figure 3c, we added

“tRHs refers to tRNA-halves and tRFs refers to tRNA-derived fragments. i-tRFs refers to internal tRNA-derived fragments.”. In the caption of Figure 5a, we defined AUC as Area Under the receiver operating characteristic Curve.

Question5: There are many descriptions for the categories of tsRNA, such as “5’-tRHs”, “i-tRFs”, “3’-tRFs” and “3’-tRHs” in the manuscript, and other abbreviations in previous papers. Meanwhile, the author should discuss the advantage and scientific merit of tsRNAs over other previously explored biomarkers. Some relevant citation could be found, "DOI:10.1155/2022/8708312 ".

Answer: We added a paragraph discussing the advantage and scientific merit of tsRNAs in the discussion section. “Recent studies have reported that tsRNAs can regulate cancer progression at the post-transcriptional level through multiple mechanisms and are thus considered as critical regulators and biomarkers of cancer. When we sorted the average specificities of different cfRNA combinations in the discovery and validation cohort, we found that tsRNAs were present in all top five combinations ("m+sn+sno+ts”, “m+mi+ts”, “m+sn+ts”, “sn+ts”, “m+ts”) (Supplementary Data 6). This finding suggested that the inclusion of tsRNAs in the combination panel significantly enhances the specificity of the machine learning models in lung cancer detection. This heightened specificity is of paramount importance in early cancer screening, where the cost and psychological implications of false positives can be substantial.”

Fu, M. *et al.* Emerging roles of tRNA-derived fragments in cancer. *Mol Cancer* **22**, 30, doi:10.1186/s12943-023-01739-5 (2023).

Liu, X. *et al.* A pro-metastatic tRNA fragment drives Nucleolin oligomerization and stabilization of its bound metabolic mRNAs. *Mol Cell* **82**, 2604-2617 e2608, doi:10.1016/j.molcel.2022.05.008 (2022).

Ren, J. et al. The tRNA-Cys-GCA Derived tsRNAs Suppress Tumor Progression of Gliomas via Regulating VAV2. Dis Markers 2022, 8708312, doi:10.1155/2022/8708312 (2022).

Question6: Minor comments: The authors should revise English writing carefully and

eliminate some errors in the paper to make the paper easier to read.

Answer: Thank you for your careful review and for pointing out the grammatical errors in our manuscript. To address these issues, we have utilized the DeepL tool (https://www.deepl.com/write) to assist in refining the English writing of our manuscript. We believe that this tool has significantly enhanced the clarity and coherence of our text. We kindly request that you refer to the tracked changes version of the manuscript, where all the modifications have been highlighted.

Other revisions we made:

1. Renamed all ‘msRNA’ to ‘mRNA’ and all ‘lsRNA’ to ‘lncRNA’ to avoid confusion.
2. Added ‘Supplementary Data 6- Summary of different cfRNA combinations’ to show AUC, sensitivities, specificities of different combinations.
3. Replaced fig. 5c. To avoid confusion between sensitivity at 100% specificity and the sensitivity/ sensitivity calculated from risk scores, we determined to use AUC as indicator of model accuracy throughout the paper instead of using sensitivity at 100% specificity.
4. Reordered supplementary fig. 12-14. Replaced the cumulative read count boxplot in supplementary fig. 14 to risk score boxplot of individuals. The presentation of the cumulative read count boxplot is less meaningful than the read count boxplot of individual cfRNA in supplementary fig. 12, and their interpretations are duplicative.

Reviewer #2

As SLiPiR-seq literally could sequence any fragmented RNA, it will bring challenge in following analysis of the sequenced data. However, these challenges could also be opportunity in developing new analysis protocols and identifying new diagnostic markers using these under investigated cfRNAs. Additionally, some minor issues in grammar need to be addressed before final acceptance as shown below.

We greatly appreciate the reviewer recognizing the novelty and potential impact of the SLiPiR-seq method. We agree that developing new analysis approaches for this novel cfRNA sequencing data represents an exciting area for future work, with potential to uncover previously underexplored RNA biomarker candidates.

We sincerely appreciate the reviewer highlighting the minor grammar issues present in the initial draft. We have thoroughly proofread the manuscript and addressed these concerns as suggested.

All changes were incorporated in the revised manuscript with track changes.

Other revisions we made:

1. Renamed all ‘msRNA’ to ‘mRNA’ and all ‘lsRNA’ to ‘lncRNA’ to avoid confusion.
2. Added ‘Supplementary Data 6- Summary of different cfRNA combinations’ to show AUC, sensitivities, specificities of different combinations.
3. Replaced fig. 5c. To avoid confusion between sensitivity at 100% specificity and the sensitivity/ sensitivity calculated from risk scores, we determined to use AUC as indicator of model accuracy throughout the paper instead of using sensitivity at 100% specificity.
4. Reordered supplementary fig. 12-14. Replaced the cumulative read count boxplot in supplementary fig. 14 to risk score boxplot of individuals. The presentation of the cumulative read count boxplot is less meaningful than the read count boxplot of individual cfRNA in supplementary fig. 12, and their interpretations are duplicative.

Reviewer #3

Question 1: To the best of my knowledge, the author is the first group that demonstrated SLiPiR-seq can capture more RNA species than conventional adapter ligation-based methods. The authors also provided extensive technical characterisation of SLiPiR-seq data through clear visualisations of sequencing metrics and comparisons with canonical methods.

However, the SLiPiR-seq methodology described in this study is not significantly different from the method previously described by Maguire et al. Both methods use splint ligation as a key step to ligate adapters to RNA fragments without requiring a 5'-phosphate group. Maguire's team compared SLiPiR-seq against Illumina's TruSeq, New England Biolab's NEBNext, Perkin Elmer's NEXTflex methods, while the current study only compared SLiPiR-seq against NEBNext.

Maguire, S., Lohman, G. J., & Guan, S. (2020). A low-bias and sensitive small RNA library preparation method using randomized splint ligation. Nucleic acids research, 48(14), e80-e80.

The method developed by Maguire et al was based on the following works.

Nilsen T. W. (2013). Splinted ligation method to detect small RNAs. Cold Spring Harbor protocols, 2013(1), pdb.prot072611. https://doi.org/10.1101/pdb.prot072611

Jin, J., Vaud, S., Zhelkovsky, A. M., Posfai, J., & McReynolds, L. A. (2016). Sensitive and specific miRNA detection method using SplintR Ligase. Nucleic acids research, 44(13), e116. https://doi.org/10.1093/nar/gkw399

Proper discussion and citation of the above works would allow readers differentiate this work from established literature. Besides, the protocol optimisations made by the authors, presumably allowing the method to be more sensitive to low quantity of cfRNA, should be clearly highlighted in the main text.

Answer: We appreciate the reviewer for raising the pertinent point regarding the use of splint ligation for library preparation in previous studies. While both SLiPiR-seq and Maguire et al.'s method utilize splint ligation as a central step for adapter ligation, it is crucial to delineate the specific distinguishing features between these two approaches.

Firstly, in the method proposed by Maguire et al., they optimized their workflow to ligate a 5'-splint adapter containing six degenerate nucleotides to the 5' end of RNA prior to reverse transcription. This approach effectively enhances ligation efficiency compared to traditional single-strand RNA-RNA base adapter ligation, thereby increasing sensitivity, especially for low-input RNA libraries

(shown below). However, it's noteworthy that even in this method, 5'-phosphate modification of RNA remains a prerequisite for successful adapter ligation.

In contrast, SLiPiR-seq introduces several distinctive attributes (shown below):

1) Reverse transcription prior to ligation: In SLiPiR-seq, reverse transcription is performed as the initial step. Important sequencing elements such as sample barcode and the P7 primer sequences are added to the first-strand cDNA after reverse transcription.

2) DNA-based splint adapter: SLiPiR-seq employs a DNA-based splint adapter that is capable of ligating to the 3' end of the first-strand cDNA, corresponding to the 5' end of the RNA before reverse transcription. Consequently, it circumvents the necessity for a 5'-phosphate modification on the RNA, ensuring compatibility with RNA molecules lacking this modification.

To address the reviewer's valid concern and highlight these distinctions, we have incorporated a discussion within the manuscript, supplemented by appropriate citations of the relevant works by Maguire et al. (2020) and Nilsen (2013).

“The use of splint ligation for small RNA library preparation has been reported in previous studies. It is crucial to delineate the specific distinguishing features between SLiPiR-seq and the previous approach. Maguire et al. employed a novel RNA splint ligation adapter containing six degenerate nucleotides at the 5' end of the RNA. This approach effectively increases ligation efficiency compared to traditional single-stranded RNA-RNA base adapter ligation, thereby increasing sensitivity, especially for low-input RNA libraries. However, it's noteworthy that even with this method, 5'-phosphate modification of the RNA remains a prerequisite for successful adapter ligation. In SLiPiR-seq, reverse transcription is performed prior to adapter ligation. Important sequencing elements such as sample barcode and the P7 primer sequences are added to the first-strand cDNA after reverse transcription. SLiPiR-seq employs a DNA-based splint adapter that is capable of ligating to the 3' end of the cDNA, corresponding to the 5' end of the RNA. Consequently, this bypasses the necessity for a 5'-phosphate modification on the RNA.”

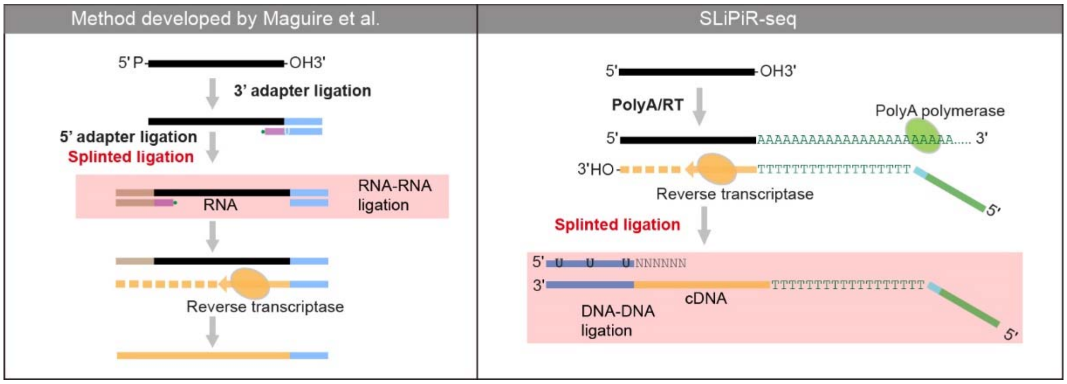

Comparison of the splint ligation strategies utilized in the method developed by Maguire et al. and SLiPiR-seq.

Question 2: In line 115, the authors noted that "the fragment size of mRNAs and lncRNAs exhibited smoother distribution and broader coverage (23-60 nt)". Could the authors apply appropriate statistical tests to substantiate the claim?

Answer: We appreciate the reviewer raising this important point about substantiating our claim regarding the fragment size distribution and coverage of mRNAs and lncRNAs. To statistically support this observation, we conducted further analysis using the median and median absolute deviation (MAD) to characterize the distribution of fragment sizes between the NEBNext and SLiPiR-seq datasets as shown below (Table 1).

Table1 Statistical comparison of mRNA and lncRNA fragment size profiles detected by NEBNext and SLiPiR-seq.

	NEBNext	SLiPiR-seq
mRNA	31bp ± 0bp	36bp ± 14.8bp
lncRNA	31bp ± 8.9bp	33bp ± 11.9bp

In summary, the larger median fragment size values combined with the greater MAD provide statistical evidence that SLiPiR-seq detected mRNAs and lncRNAs with a smoother distribution and broader size range compared to NEBNext. We have incorporated these quantitative results in the revised manuscript to support our initial qualitative observation.

Question 3: In line 170, the authors noted "a significant increase in the cumulative expression level in early stage LC patients compared to controls". Could this observation due to confounding factors, bias in normalisation or multi-mapped reads? A recent study found that

common normalization methods vary across different datasets.

Düren, Y., Lederer, J., & Qin, L. X. (2022). Depth normalization of small RNA sequencing: using data and biology to select a suitable method. Nucleic acids research, 50(10), e56. https://doi.org/10.1093/nar/gkac064

Answer: We greatly appreciate the reviewer's insightful inquiry into the critical issue of data normalization in small RNA sequencing studies and their reference to the work of Düren et al. (2022) on depth normalization in small RNA sequencing. Normalization methods play a crucial role in accurately characterizing gene expression, and we acknowledge the potential impact of confounding factors, normalization biases, and multi-mapped reads in our analysis.

At the outset of our study, we recognized the significance of choosing an appropriate normalization method for our cfRNA data. Given that plasma cfRNA are small RNA fragments, and not subjected to manual fragmentation during library preparation, we hypothesized that Reads Per Million (RPM) normalization would be a suitable choice. To validate this hypothesis, we conducted rigorous validation experiments using the gold-standard technique for RNA quantification. We profiled 181 distinct plasma cfRNAs and compared their expression levels normalized by RPM (mapped reads as indicator of RPM) to quantitative real-time PCR (qPCR) results. We observed a strong overall concordance (R=0.86) between RPM-normalized expression levels and Ct values measured by qPCR (Fig. 2a). This high concordance was consistently observed for various RNA species, including lncRNA (R=0.83), miRNA (R=0.81), piRNA (R=0.71), mRNA (R=0.87), snRNA (R=0.89), rsRNA (R=0.95), snoRNA (R=0.82), ysRNA (R=0.97), and tsRNA (R=0.85) (Supplementary Fig5).These findings provide strong evidence for the reliability of RPM normalization in our study.

To further address concerns related to potential normalization bias within cohort-based study utilizing SLiPiR-seq, we conducted correlation analysis on cfRNA profiles from cancer patients and cancer-free individuals within our dataset using RPM normalization method. This analysis revealed a high overall correlation (R=0.987, p<2.2e-16) between the two groups (Fig. 4a), further supporting the robustness and consistency of the normalization method across different sample sets.

Concerning the issue of multi-mapped reads, we implemented specific measures to mitigate potential biases. For RNA species such as rsRNA, ysRNA, and tsRNA, we utilized a custom Python script designed for sequence-based exact matching to minimize any potential bias introduced by multiple-mapping issues (Fig. 3a and Supplementary Fig. 1). For miRNA and piRNA, we employed the bowtie2 mapping algorithm with stringent parameters to reduce the presence of multi-mapped reads (Supplementary Fig. 1). Furthermore, for mRNA, lncRNA, snoRNA, and snRNA, we

implemented a pre-processing step to generate a distinct Gene Transfer Format (GTF) annotation file for each RNA species, facilitating precise annotation of genomic locations. We then adopted strand-specific (-s, 1) read calling strategies in FeatureCounts, ensuring the accurate assignment of reads to their respective RNA species and strands (Supplementary Fig. 1). These efforts have significantly reduced the incidence of multi-mapped rates.

In conclusion, we consider that the observed increase in cumulative expression levels in early stage LC patients is primarily attributed to the upregulated expression of specific cfRNAs in cancer patients. Despite the challenges associated with data normalization, our comprehensive validation experiments and correlation analyses support the reliability and accuracy of our normalization process. This allows us to interpret the observed differences in cfRNA expression between groups as indicative of biological variations associated with lung cancer.

Question 4: In line 185-261, the authors attempted to use SLiPiR-seq data to develop a cfRNA signature for early detection of cancer. However, given by the relatively small sample size and diverse cancer types, it is unclear if the study has enough statistical power to substantiate the claim in line 260. "Collectively, these results demonstrate that cfRNA signatures identified by SLiPiR-seq can precisely discriminate different cancer types".

Answer: We updated the conclusion of this section into "Collectively, these results demonstrate that cfRNA signatures identified by SLiPiR-seq <u>are promising in the classification of</u> different cancer types, motivating us to validate the clinical utility of SLiPiR-seq in larger sample cohorts in future investigations."

Question 5: In Fig. 2a, it looks like the dots corresponding to miRNAs are all below the trend line. Could it mean that miRNA expression is most likely underestimated by SLiPiR-seq?

Answer: We appreciate the reviewer for bringing up this important observation. Upon careful reevaluation of our data, we have identified an error in placing the trendline in the original presentation of Fig. 2a. We have since corrected the trendline to accurately represent the data.

Furthermore, to address the concern regarding the potential underestimation of miRNA expression by SLiPiR-seq, we conducted a thorough analysis using the data provided in Supplementary Data 1. We specifically focused on the relationship between Ct values and log2RPM for miRNA detection, as well as the comparison of miRNA expression with that of other RNA species. Our analysis revealed that when considering only miRNAs, there are 31 data points located above the trendline and 41 below it. In contrast, when analyzing the combined expression of the other 8 RNA

species alongside miRNAs, there are 22 data points above and 50 below the trendline. These findings indicate that relative to the other RNA species, miRNA expression might be slightly underestimated by SLiPiR-seq.

It is important to emphasize that while there might be a slight underestimation of miRNA expression relative to other RNA species, this observation does not compromise the integrity of our downstream differential analysis. Specifically, our analysis methodology does not involve the direct comparison of the abundance of each RNA species within the cfRNA repertoire detected by SLiPiR-seq. Instead, we focus on the relative expression of individual miRNAs, which we have found to be highly correlated with qPCR results.

Question 6: In line 140, the authors classified tsRNAs, rsRNAs and ysRNAs species based on the first 15 nucleotides. I would expect more comprehensive comparison of RNA species using established databases such as Rfam and RNAcentral.

Answer: The other reviewer also pointed out a question about the classification of RNA species on the feedback: “It is interesting, read counts of all subspecies was summed to represent the counts of one RNA species. But, sometimes, the length of subspecies fragments varies between 16bp and 50bp in one RNA species (Supplementary Data 3), this issue is worth thinking about deeply.”

To address this issue, we removed the analytical step that classifies fragments with identical sequences for the first 15 nucleotides from the 5' end as one RNA species. To avoid too many RNA species, we also tweaked the codes for bowite2 alignment to tolerate zero mismatch. As a result, we detected a total of 45397 rsRNA and 2664 ysRNA unique sequences.

However, the revision on the classification of RNA species inevitably altered other results demonstrated in the paper, resulting in extensive modifications. For example, the number of detected RNA species for tyr-sRNA in fig. 1c and fig. 2d were updated. Fig. 3a was also updated.

After revision, a total of 152858 cfRNA species were used for the differential expression analysis, which is 1.5 times more than before. This will affect the normalization of the cfRNA read count matrix and the calculation of adjusted p values during the DESeq2 analysis. Therefore, all results from fig. 4 to fig. 6 and their supplementary figures were inevitably changed. After revision, rsRNA accounts for 73.4% of all differentially expressed cfRNA and overshadows the performance of other RNA types. To compensate for this issue, we adjusted the inclusion criteria of candidate cfRNA selection from log2 fold change>1 to >0.8, which allows more cfRNAs of other types to be applied for the subsequent feature selection test.

Overall, although most results were updated, the inclusion of more tyr-sRNA species does not alter the findings and conclusions of our study. Interestingly, LR and SVM models training with 50 rsRNA (SVM AUC= 0.819 [0.786-0.843]) or 25 ysRNA (SVM AUC= 0.793 [0.747-0.829]) features showed high AUC in LC detection. However, models trained by the RF algorithm shared no agreement with these results (Supplementary Fig. 11c). Therefore, rsRNA and ysRNA were not used in the combination test. Again, the five RNA types of interest (mRNAs, miRNAs, snRNAs, snoRNAs, and tsRNAs) were used in the combination test and the cancer classification test.

Back to this question, Rfam and RNAcentral are well-known and established databases, and they have comprehensive information about tRNAs, rRNAs and yRNAs. However, they do not contain information about tsRNAs, rsRNAs and ysRNAs. Therefore, we are not able to conduct comparison of these small RNAs using Rfam and RNAcentral.

Question 7: In Fig. 4e, the legends are unclear. The colour blocks below the dendrogram, on the left of the heatmap, at the bottom of the heatmap should be clearly labelled. Description of blocks in grey is not defined.

Answer: We added 'left', 'top' and 'bottom' on the figure legend of fig. 4e to make the color blocks clear. We also removed the gray color from cancer-free controls for the bottom color block.

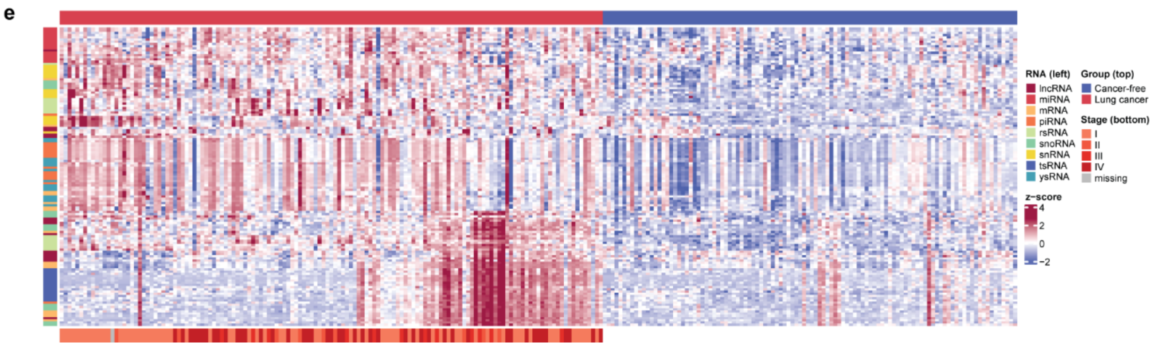

Question 8: In line 168, should absolute log2 fold change < 1 be used instead?

Answer: It depend very much on what we are looking for in the study. If we are screening for internal controls for qPCR quantification, absolute log2 fold change < 1 should be used. On the other hand, if we aim to identify cancer biomarkers, we believe that log2 fold change > 1 should be considered. This is because the detection of cfRNA signatures with a larger fold change between cases and controls will be easier in a cancer detection test.

Question 9: The surprisingly high sensitivity of 96.15% and specificity of 100% may indicate overfitting under high dimensionality of the features. While it may not be feasible to have independent training, testing and validation samples, the use of regularisation techniques and cross-validation may alleviate overfitting of features.

Answer: We agree with the reviewer's concern about the overfitting of machine learning models in the validation cohort. In the original submission, while we applied regularization and cross-validation in our models, we selected the cfRNAs with the highest AUC in the validation cohort among three different feature selection methods for the combination of multiple types of cfRNAs, which may result in overfitting of the model in the validation cohort and compromising the purpose of validation. In our revision, we used cfRNAs with the highest AUC in the test set of the discovery cohort among three different feature selection methods for the combination test. The validation process of the combination test is now unbiased. After revision, the best combination ("m+sn+sno+ts" panel) shows 100% specificity and 99.28% sensitivity in the discovery cohort and 95.24% specificity and 76.92% sensitivity in the validation cohort.

Other revisions we made:

1. Renamed all 'mRNA' to 'mRNA' and all 'lncRNA ' to 'lncRNA' to avoid confusion.
2. Added 'Supplementary Data 6- Summary of different cfRNA combinations' to show AUC, sensitivities, specificities of different combinations.
3. Replaced fig. 5c. To avoid confusion between sensitivity at 100% specificity and the sensitivity/ sensitivity calculated from risk scores, we determined to use AUC as indicator of model accuracy throughout the paper instead of using sensitivity at 100% specificity.
4. Reordered supplementary fig. 12-14. Replaced the cumulative read count boxplot in supplementary fig. 14 to risk score boxplot of individuals. The presentation of the cumulative read count boxplot is less meaningful than the read count boxplot of individual cfRNA in supplementary fig. 12, and their interpretations are duplicative.

REVIEWERS' COMMENTS

Reviewer #1 (Remarks to the Author):

I am satisfied with the Author's response as well as the revision of this manuscript, and have no more comments.

Reviewer #2 (Remarks to the Author):

The authors have addressed all my concerns. This work disclosed the necessity of exploring multiple species of RNAs in plasma as biomarkers for early-detection of cancer. I recommend publication of the corrected manuscript in Nature Communications.

Reviewer #3 (Remarks to the Author):

Thanks for addressing the comments in my first review. Please refer to my further comments below:

Qs1: No further comments

Qs2: Median and median absolute deviation are not good parameters to substantiate if a distribution is smoother and broader. Please consider using a Goodness-of-Fit test to check the smoothness of a distribution. Meanwhile, kurtosis is a common measure of the breadth (tailedness) of a distribution.

Qs3: The authors showed that the RPM values correlates with qPCR Ct values, which supports the reliability of RPM normalisation. Ideally, artificial spike-ins that corresponds to the cell number or DNA quantity can be used to measure if a global difference in cumulative expression level exists in future studies.

The authors further mentioned the use of sequence-based exact matching and stringent mapping parameters to reduce the chance of multi-mapping. Since some RNA species have very similar sequences (e.g. miRNA), these filters may affect the sensitivity of detection. Is there any figure or table that summarises the impact of these filtering strategies on the number of detected RNA species?

Qs4: No further comments

Qs5: What are the potential reasons that might lead to the underestimation of miRNA expression by SLiPiR-seq? In relation to my comments in Qs3, could the stringent filtering strategies affect the sensitivity of counting miRNAs?

Qs6: No further comments
Qs7: No further comments
Qs8: No further comments

Qs9: Please show the number of samples in the discovery, testing, and validation cohort. Besides, please indicate the cohort splitting strategy in detail, such as whether random sampling or stratified sampling were used.

RESPONSE TO REVIEWERS' COMMENTS

Reviewer #3

Thanks for addressing the comments in my first review. Please refer to my further comments below:

Qs1: No further comments

Qs2: Median and median absolute deviation are not good parameters to substantiate if a distribution is smoother and broader. Please consider using a Goodness-of-Fit test to check the smoothness of a distribution. Meanwhile, kurtosis is a common measure of the breadth (tailedness) of a distribution.

Answer: We appreciate the insightful suggestion from the reviewer regarding the statistical analysis of fragment size distribution. In response to this, we conducted a comprehensive analysis of the mRNA and lncRNA fragment size distributions, fitting them to the Gamma distribution and calculating the associated α and β parameters. The obtained results contribute valuable insights into the smoothness and breadth of these distributions.

The fragment size distributions for mRNAs in both NEBNext (α=3.32, β=0.10) and SLiPiR-seq (α=10.22, β=0.24) were scrutinized, revealing a difference in smoothness, with SLiPiR-seq exhibiting a smoother distribution. A similar trend was observed in lncRNA fragment size distributions, where SLiPiR-seq (α=12.07, β=0.35) demonstrated a smoother distribution compared to NEBNext (α=6.29, β=0.21).

Additionally, we utilized kurtosis as a measure of the breadth or tailedness of the fragment size distribution. The calculated kurtosis values for mRNA fragments indicated a broader distribution in SLiPiR-seq (11.25) compared to NEBNext (13.22). Similarly, for lncRNA fragments, SLiPiR-seq (14.92) exhibited a broader distribution than NEBNext (30.58).

In conclusion, the assessment of the fragment size distribution, incorporating both α and β parameters from the Gamma distribution fitting and kurtosis values, indicates that SLiPiR-seq produces smoother and broader mRNA and lncRNA fragment distributions. We have incorporated these refined analyses into the main manuscript as per the reviewer's recommendations to provide a more accurate comparison of fragment size distributions.

Qs3: The authors showed that the RPM values correlates with qPCR Ct values, which supports the reliability of RPM normalisation. Ideally, artificial spike-ins that corresponds to the cell number or DNA quantity can be used to measure if a global difference in cumulative expression level exists in future studies.

The authors further mentioned the use of sequence-based exact matching and stringent mapping parameters to reduce the chance of multi-mapping. Since some RNA species have very similar sequences (e.g. miRNA), these filters may affect the sensitivity of detection. Is there any figure or table that summarises the impact of these filtering strategies on the number of detected RNA species?

Answer: We appreciated the valuable suggestion made by the reviewer regarding the utilization of artificial spike-ins for more accurate quantification of SLiPiR-seq results. This recommendation has been duly noted, and we plan to incorporate this experimental approach in future studies. The incorporation of artificial spike-ins is anticipated to provide enhanced precision in evaluating detection accuracy and further enable the assessment of global differences in cumulative expression levels.

For the mapping issues, we have implemented sequence-based exact matching strategies for rsRNA, ysRNA, and tsRNA due to their closely related reference sequences, as detailed in table1 shown below. Notably, the reference rsRNA and ysRNA sequences were derived from the SAM file, which was generated through mapping the clean reads to human rRNA or RNY genomic sequence. Therefore, the reference sequences were comprehensive, and thus minimize the risk of missed or inaccurately detected RNA species. Similarly, tsRNA data were obtained from MINTbase, known for its exhaustive coverage of all tsRNA sequences, further mitigating the possibility of misdetection.

Table 1 Reference sequence of representative rsRNA, ysRNA, and tsRNA.

ID	Sequence
rsRNA：	
Homo-18S-8780	TAACAAGGTTTCCGTAGGTGAACCTGCGGAAGGATCATT
Homo-18S-5733	GTAACAAGGTTTCCGTAGGTGAACCTGCGGAAGGATCATT
Homo-18S-4933	CGTAACAAGGTTTCCGTAGGTGAACCTGCGGAAGGATCATT
Homo-18S-4893	TCGTAACAAGGTTTCCGTAGGTGAACCTGCGGAAGGATCATT
Homo-18S-16704	GTCGTAACAAGGTTTCCGTAGGTGAACCTGCGGAAGGATCATT
Homo-18S-6367	AGTCGTAACAAGGTTTCCGTAGGTGAACCTGCGGAAGGATCATT
ysRNA:	
RNY4-21	CCCACTGCTAAATTTGACTGGCTT
RNY4-9	CCCCACTGCTAAATTTGACTGGCTT
RNY4-16	CCCCCACTGCTAAATTTGACTGGCTT

RNY4-33	CCCCCCACTGCTAAATTTGACTGGCTT
RNY4-177	ACCCCCCACTGCTAAATTTGACTGGCTT
RNY4-433	AACCCCCCACTGCTAAATTTGACTGGCTT
tsRNA:	
tRF-17-BDHPIWL	AAAACATCAGATTGTGA
tRF-18-BDHPIWD6	AAAACATCAGATTGTGAA
tRF-19-BDHPIWH7	AAAACATCAGATTGTGAAT
tRF-20-BDHPIWOM	AAAACATCAGATTGTGAATC
tRF-21-BDHPIWOME	AAAACATCAGATTGTGAATCT
tRF-22-BDHPIWOMP	AAAACATCAGATTGTGAATCTG

In contrast, miRNA detection is rely on the reference sequences from miRbase, which cannot be used to do exact sequence-based matching due to the inherent limitation of not covering all possible sequences generated through sequencing. To address this, a two-step filtering process was implemented in this study. The first step excluded fragments shorter than 19 nucleotides, followed by mapping using bowtie2 with default parameters. Subsequently, only miRNA reads exhibiting an exact match without insertions, deletions, or soft/hard clipping were included, ensuring accurate miRNA counting. This stringent approach aimed to distinguish miRNA from tsRNA, given their potential sequence similarities. Specific examples, such as the similarity between has-miR-1260b and tRF-17-HR0VX6J, hsa-miR-7977 and tRF-17-YR66EFJ, and hsa-miR-4286 and tRF-17-0RER9LJ, were highlighted to underscore the necessity of the filtering step in preventing potential misannotation (Table 2 shown below). The subsequent figure illustrates the impact of these filtering steps on the decrease in detected miRNA species (Fig. 1a). Notably, the majority of the omitted miRNA species exhibited either sequence similarity to tsRNA or lower expression levels.

Table 2 Comparison of representative miRNA and tsRNA with similar sequences.

ID	Sequence
tRF-17-HR0VX6	ATCCCACCGCTGCCACC
has-miR-1260b	ATCCCACCACTGCCACCAT
tRF-17-0RER9LJ	ACCCCACTCCTGGTACC
hsa-miR-4286	ACCCCACTCCTGGTACC

tRF-17-YR66EFJ	TTCCCGGCCAACGCACC
hsa-miR-7977	TTCCCAGCCAACGCACCA

Qs4: No further comments

Qs5: What are the potential reasons that might lead to the underestimation of miRNA expression by SLiPiR-seq? In relation to my comments in Qs3, could the stringent filtering strategies affect the sensitivity of counting miRNAs?

Answer: We appreciate the insightful question raised by the reviewer concerning the potential reasons for the observed underestimation of miRNA expression in SLiPiR-seq. The underestimation may indeed be attributed to the applied filtering and read count strategies, as expounded in response to Qs3. To elucidate this, a comparison of the rpm values for the Top 5 detected miRNA, both with and without the filtering steps, was conducted (Fig. 1b). Notably, a slight decrease in rpm values was observed for miRNAs detected through the filtering steps (Fig. 1b). This phenomenon may contribute to the observed underestimation of miRNA in the correlation analysis of qPCR with SLiPiR-seq results, as depicted in Fig2a within the manuscript.

A comprehensive correlation analysis was further conducted, several miRNAs with low expression or overlapping sequences with tsRNA were exclusively detected by the non-filtering process (Fig 1c). These results implies that while the filtering step could enhance the precision of miRNA annotation, it may concurrently lead to diminished detection sensitivity, as pointed out by the reviewer. Despite these observations, a robust concordance persisted between results obtained through the filtering and non-filtering strategies (R=0.96, Fig. 1c). This high degree of correlation suggests that, while the filtering steps might impact the sensitivity of miRNA detection, they do not alter the relative expression levels of individual miRNAs in plasma. Importantly, this observation underscores that the integrity of our downstream differential analysis remains uncompromised by the filtering processes.

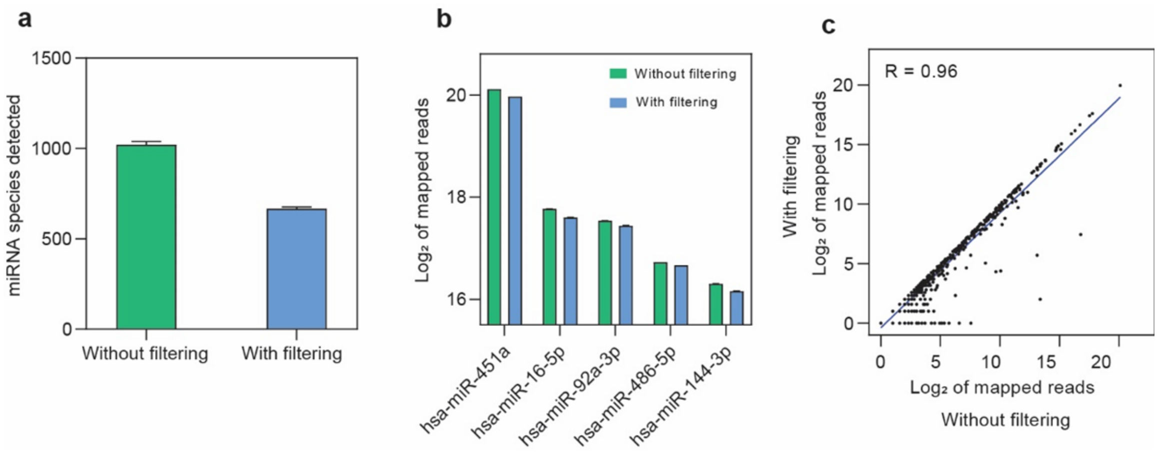

Fig. 1 The impact of filtering procedure on plasma miRNA detection.

Qs6: No further comments

Qs7: No further comments

Qs8: No further comments

Qs9: Please show the number of samples in the discovery, testing, and validation cohort. Besides, please indicate the cohort splitting strategy in detail, such as whether random sampling or stratified sampling were used.

Answer: There are 245 samples in the discovery cohort (196 samples for training and 49 samples for testing) in the lung cancer detection study. There are 382 samples in the discovery cohort (306 samples for training and 76 samples for testing) in the pan-cancer classification study. There are 53 samples in the validation cohort for both studies. The discovery cohort was randomly partitioned into training and test sets in an 80-20% manner using the createDataPartition function in the R package caret. The cohort splitting strategy was based on random sampling. The sample partitioning and model train-test processes were repeated 100 times for all machine learning analyses to avoid sampling bias.

We have added the number of samples in each cohort in the “Method - Sample grouping and partitioning” section of our manuscript: “In the lung cancer study, samples from NOR_SZBA and LC_SZDE were grouped as a discovery cohort (N=245). In the pan-cancer study, samples from NOR_SZBA, LC_SZDE, BRC_NXYK, CRC_NXYK, GC_NXYK and HCC_NXYK were grouped as a discovery cohort (N=382). Samples from NOR_SZDW and LC_SZBU were grouped

as a validation cohort (N=53) to validate models established in both the lung cancer detection study and the pan-cancer classification study.”
